# The characterization of key physiological traits of medicinal cannabis (*Cannabis sativa L.*) as a tool for precision breeding

**DOI:** 10.1186/s12870-021-03079-2

**Published:** 2021-06-26

**Authors:** Erez Naim-Feil, Luke W. Pembleton, Laura E. Spooner, Alix L. Malthouse, Amy Miner, Melinda Quinn, Renata M. Polotnianka, Rebecca C. Baillie, German C. Spangenberg, Noel O. I. Cogan

**Affiliations:** 1 Agriculture Victoria, AgriBio, Centre for AgriBioscience, Bundoora, VIC 3083 Australia; 2 School of Applied Systems Biology, La Trobe University, Bundoora, VIC 3086 Australia

**Keywords:** Yield, Flowering time, Correlations, Growth rate, Heritability, Selection, Prediction equation

## Abstract

**Background:**

For millennia, drug-type cannabis strains were extensively used for various medicinal, ritual, and inebriant applications. However, cannabis prohibition during the last century led to cultivation and breeding activities being conducted under clandestine conditions, while scientific development of the crop ceased. Recently, the potential of medicinal cannabis has been reacknowledged and the now expanding industry requires optimal and scientifically characterized varieties. However, scientific knowledge that can propel this advancement is sorely lacking. To address this issue, the current study aims to provide a better understanding of key physiological and phenological traits that can facilitate the breeding of advanced cultivars.

**Results:**

A diverse population of 121 genotypes of high-THC or balanced THC-CBD ratio was cultivated under a controlled environment facility and 13 plant parameters were measured. No physiological association across genotypes attributed to the same vernacular classification was observed. Floral bud dry weight was found to be positively associated with plant height and stem diameter but not with days to maturation. Furthermore, the heritability of both plant height and days to maturation was relatively high, but for plant height it decreased during the vegetative growth phase. To advance breeding efficacy, a prediction equation for forecasting floral bud dry weight was generated, driven by parameters that can be detected during the vegetative growth phase solely.

**Conclusions:**

Our findings suggest that selection for taller and fast-growing genotypes is likely to lead to an increase in floral bud productivity. It was also found that the final plant height and stem diameter are determined by 5 independent factors that can be used to maximize productivity through cultivation adjustments. The proposed prediction equation can facilitate the selection of prolific genotypes without the completion of a full cultivation cycle. Future studies that will associate genome-wide variation with plants morphological traits and cannabinoid profile will enable precise and accelerated breeding through genomic selection approaches.

**Supplementary Information:**

The online version contains supplementary material available at 10.1186/s12870-021-03079-2.

## Background

*Cannabis sativa L.* (cannabis) is one of the primaeval Euro-Asian domesticated crops [1–3] that for millennia was used by humans for its fibre and oil-seeds benefits, as hemp, and for its unique resin as cannabis. Due to its multi-purpose nature, cannabis was dispersed by humans from its geographic origins into abundant locations across the globe [1, 4, 5]. During its cultivation, cannabis was adapted to address a varied range of desirable products under diverse environments that lead to a selection of several morphotypes and many genotypes originating from its monotypic genus [6–8].

Archaeological evidence for the extensive use of cannabis for pharmacological, ritual and recreational usage can be found throughout the old world [2, 7, 9, 10]. Additionally, the medicinal application of cannabis which has been widely documented for the last 3000 years, suggests treatments for a multitude of medical conditions [11–13]. The medicinal potency of cannabis is primarily attributed to the plant’s secondary metabolites, termed phytocannabinoids (cannabinoids). These cannabinoids are terpenophenolic compounds which are prevalently synthesised and stored in a resinous form over inflorescences and bracts of pistillate plants [14–16]. To date, ~ 120 cannabinoids have been scientifically recognized [17] and a number of them have been specifically linked with the treatment of particular medical disorders [18] such as Epilepsy and Parkinson’s disease [19, 20], as well as for cancer pain management [21] and as an appetite stimulant [22]. However, regardless of its popular historical medicinal application, due to its inebriant effect and drug-type classification, cannabis has been prohibited in most countries during the last century [23]. As a result, its scientific development ceased and its cultivation and breeding initiatives were required to be conducted in a clandestine manner [2, 24–26]. These breeding attempts necessitated physiological adaptations [2] and created vigorous varieties which were prosperous under indoor environment conditions [5, 16]. In addition to these adjustments, the cannabis industry was dominated by recreational consumers and therefore, breeders were also required to consistently enhance the plant's psychoactive cannabinoid- Δ9-tetrahydrocannabinol (THC) [27–29].

Despite the lack of scientific means to profile and quantify the plant’s medicinal agents (cannabinoids), cannabis breeding goals continued to be progressively implemented, with breeder’s considerations founded mainly on perceived and sensorial parameter estimations such as inebriant potency, yield production and preference for certain aromatic blends [30, 31]. Using this approach, cannabis breeders had a dramatic effect on shaping the physiological and chemical profile of recreational cultivars, as evidenced by the significant growth of THC content in cannabis plants over the last few decades [32–35] and the continued popularity of indoor cultivation methods for recreational consumption [5, 36]. As such, the focus on some other important parameters such as the non-intoxicant cannabinoid content and disease-resistance attracted very limited interest. This has led to genetic drift in many modern cannabis lines, which has attenuated the genetic diversity of contemporary cultivars [28, 37] and left invaluable genetic resources for medicinal applications untapped.

In recent years, an increasing number of published reports have provided evidence for the vast medical potential of cannabis (reviewed by Nahtigal et al., 2016, [18]). As a result, there is growing interest in understanding and advancing the latent medicinal potential of the cannabis genus [23]. Nowadays, the rapidly growing medicinal cannabis industry requires the development of optimal and scientifically characterized varieties that can be commercially utilized for medicinal applications [27]. However, only a small number of regulated drug-type cannabis breeding programs have currently been initiated by legalized growers and commercial companies [1]. Additionally, while advances in science and technology have improved many important crops over recent decades, cannabis, due to its illicit status, has not been evaluated with contemporary technologies and precise scientific methods [34, 38]. Therefore, as the need for medicinal cannabis grows, so does the necessity for scientifically based breeding programs. In order to facilitate and advance these programs, a better understanding of the biological and physiological features of the cannabis plant is required [39].

As cannabis cultivation under controlled environment (CE) facilities has a high daily operational cost [40, 41], quantifying key physiological parameters such as; the heritability of critical production traits, the association between traits and plant growth patterns including the determination of the vegetative growth stage duration prior to floral induction is essential for the optimization of medicinal cannabis cultivation and its industry development. Precision breeding for these traits for indoor cultivation can improve plant productivity, shorten the duration of each crop cycle and optimize the ratio between vegetative and reproductive cultivation stages. Despite this, extensive physiological research that can support advanced breeding initiations has been largely limited to the broadacre hemp industry as exemplified by Salentijn et al. (2015) [42].

In recent years, cannabis research has been predominantly focused on the genetic control of cannabinoids biosynthesis [43–46] and the association between environmental factors, physiological traits and the plant's secondary metabolites profile [47–49]. The knowledge elicited in these studies can be used to improve production protocols and facilitate more accurate and precise breeding. Recently, cannabis studies have emerged which do not focus directly on cannabinoid content, but rather evaluate the effect of environmental factors such as fertilization regime [50, 51] and stress conditions [52, 53] on several parameters of plant development as well as cannabinoids biosynthesis. However, while the findings of these studies were often linked back to the plant’s cannabinoids content, they did not focus specifically on the associations between key physiological traits. Therefore, while there has been considerable work conducted into cannabinoid biosynthesis, there has been limited research into developing a comprehensive index of physiological traits with a high breeding value.

The research objective of the current study was to generate scientifically based knowledge that can support the development of precision breeding tools and facilitate the generation of superior varieties for medicinal applications. To address this objective, the current study aimed to (I) examine the relationship between cannabis physiological and phenological traits, (II) quantify growth parameters under vegetative and reproductive cultivation regimes, (III) examine the heritability of high-valued production traits of cannabis and (IV) generate selection tools that can accelerate breeding initiatives.

## Results

### Phenotypic diversity

**Fig. 1:**
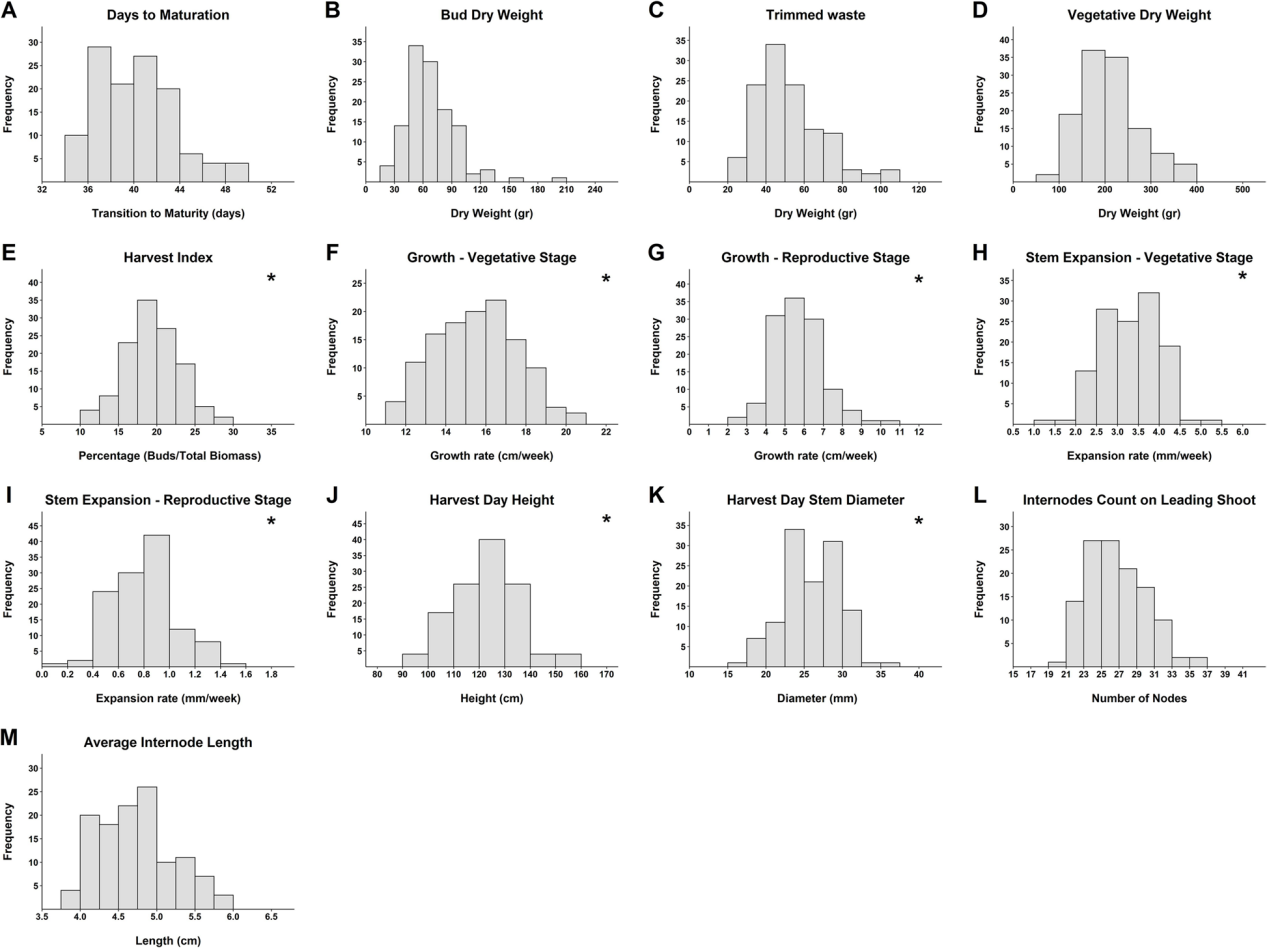
Frequency distribution of 121 cannabis lines across 13 physiological parameters. **A** Days to maturation (DTM), **B** Floral bud dry weight (BDW), **C** Trimmed waste (TW), **D** Vegetative dry weight (VDW), **E** Harvest index (HI), **F** Height growth rate during the vegetative stage (GR-V), **G** Height growth rate during the reproductive stage (GR-R), **H** Stem diameter growth rate during the vegetative stage (SDGR-V), **I** Stem diameter growth rate during the reproductive stage (SDGR-R), **J** Plant height in harvest day (PH), **K** Final measurement of stem diameter (SD), **L** Internodes count on the leading branch (IC), **M** Average internode length (IL). Normally distributed charts are marked with an asterisk on the top right corner

Frequency distributions for all of the genotypes across the 13 recorded parameters (Fig. 1) identified that the majority are normally distributed (7 out of 13). Those which were not, were often characterized by a small collection of extreme outliers (such as depicted in Fig. 1A, B, C) and in the majority of cases, the removal of these outliers restored normality to the distribution. The critical industry trait of days to maturation (DTM, Fig. 1A) ranged in the population between 34–50 days, showing that some genotypes reach the maturation transition in 68% of the time compared to others. There was also extensive phenotypic variation in floral bud (bud) dry weight (BDW), which ranged between 15 – 210 gr. per plant and the plant’s harvest index (HI) which ranged between 10%—30% (Fig. 1B and E respectively). A comparison in the genotypic growth rate between the different cultivation phases is characterized by a deceleration in plant development in response to the transition into the reproductive stage (Fig. 1F-I). Over this transition, the ranges of plant height and stem diameter growth rate reduce from 11—21 (cm*week^−1^) to 2—11 (cm*week^−1^) and from 1—5.5 (mm*week^−1^) to 0—1.6 (mm*week^−1^), respectively. Across all genotypes, the mean plant height and stem diameter growth rate decreased by 64% and 76%, respectively in response to short-day induction.

### Variation in growth patterns

**Fig. 2:**
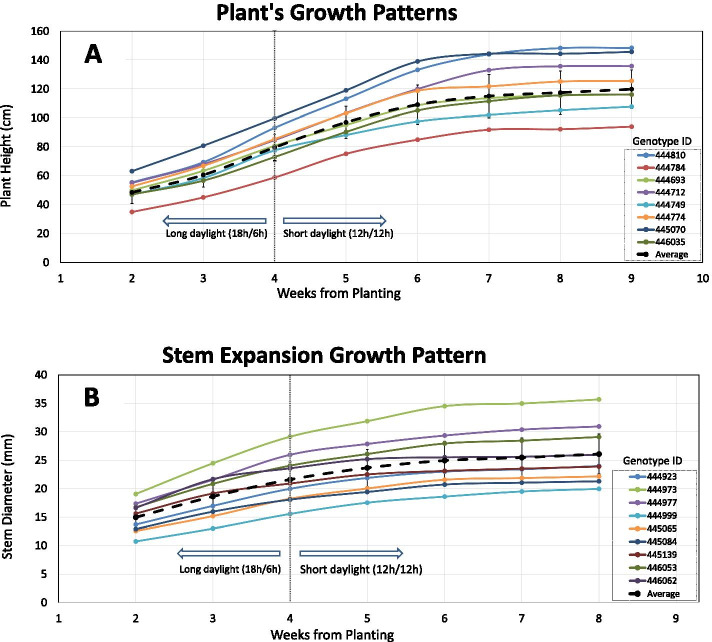
Plant growth patterns during the vegetative stage (long daylight: first 4 weeks from planting) and reproductive stage (short daylight: 4 weeks from planting onwards). The broken black line represents the mean growth pattern of all examined genotypes. Coloured lines represent the variations in growth patterns across selected genotypes. **A** Demonstrates the plant’s height growth pattern during the first 9 weeks of cultivation. **B** Shows the stem expansion growth pattern during the first 8 weeks of cultivation

Plant height (PH) and stem diameter growth patterns differed between genotypes and a varied response in vigour was observed over the weekly measurements during the cultivation period. PH and stem diameter growth patterns between genotypes were characterized by a varied response to the short daylight induction (Fig. 2). For example, variation within the response time to the reproductive stage induction was observed to differ between genotypes 444774 (duration of 2 weeks) and 444712 (duration of 3 weeks). In addition, genotypic variation with regards to growth deceleration intensity was demonstrated by the growth trends of genotypes 444749 and 445070 (as shown in Fig. 2A), while phenotypic variation was also observed regarding the timing of growth cessation, for example between genotypes 444810 (4 weeks) and 445070 (2 weeks).

The effect of the reproductive stage transformation on plant growth trends is expressed by a reduction in growth vigour which occurred within 2 weeks of the short daylight induction. Moreover, a complete PH and stem diameter growth cessation for most genotypes was observed 3 and 2 weeks, respectively, after the short daylight was induced (Fig. 2).

### Trait correlation and association

**Fig. 3:**
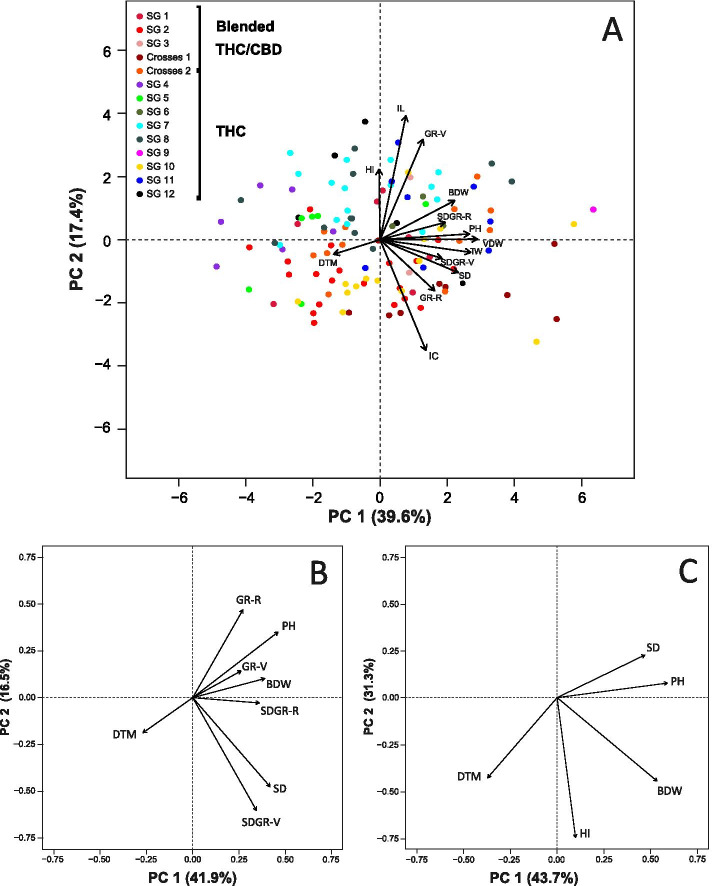
Principle component analysis (PCA) for 121 cannabis lines. **A** Demonstrates the relationship between 13 physiological traits. Colours indicate plants vernacular classification according to strain groups association (for example, “Purple Kush” or “LA Confidential” strains). Genotypes marked in red (or a variation of red colours) reflect strains with blended THC/CBD ratio while all other colour classify genotypes containing THC and no CBD (cannabinoids profile was estimated by DNA markers). **B** Shows the associations between growth parameters, DTM and BDW. **C** Presents the relationship between traits with high breeding values (DTM, HI, BDW, PH, SD)

The principal component analysis (PCA, Fig. 3) demonstrates the relationship between 13 physiological and phenological parameters alongside the performance of all genotypes. The two main PCs defined 57% of the total phenotypic variation (39.6% and 17.4%) across the examined parameters (Fig. 3A). Interestingly, no clustering based on the naming groups was apparent. The distribution of traits presented in Fig. 3A indicates that DTM is discrete from all other parameters. Moreover, the yield component BDW, is positively correlated with stem diameter growth rate during the reproductive stage (SDGR-R) and PH but contrary to this, HI, internodes length (IL) and plant height growth rate during the vegetative stage (GR-V) parameters are found to be negatively correlated with internodes count (IC).

**Fig. 4:**
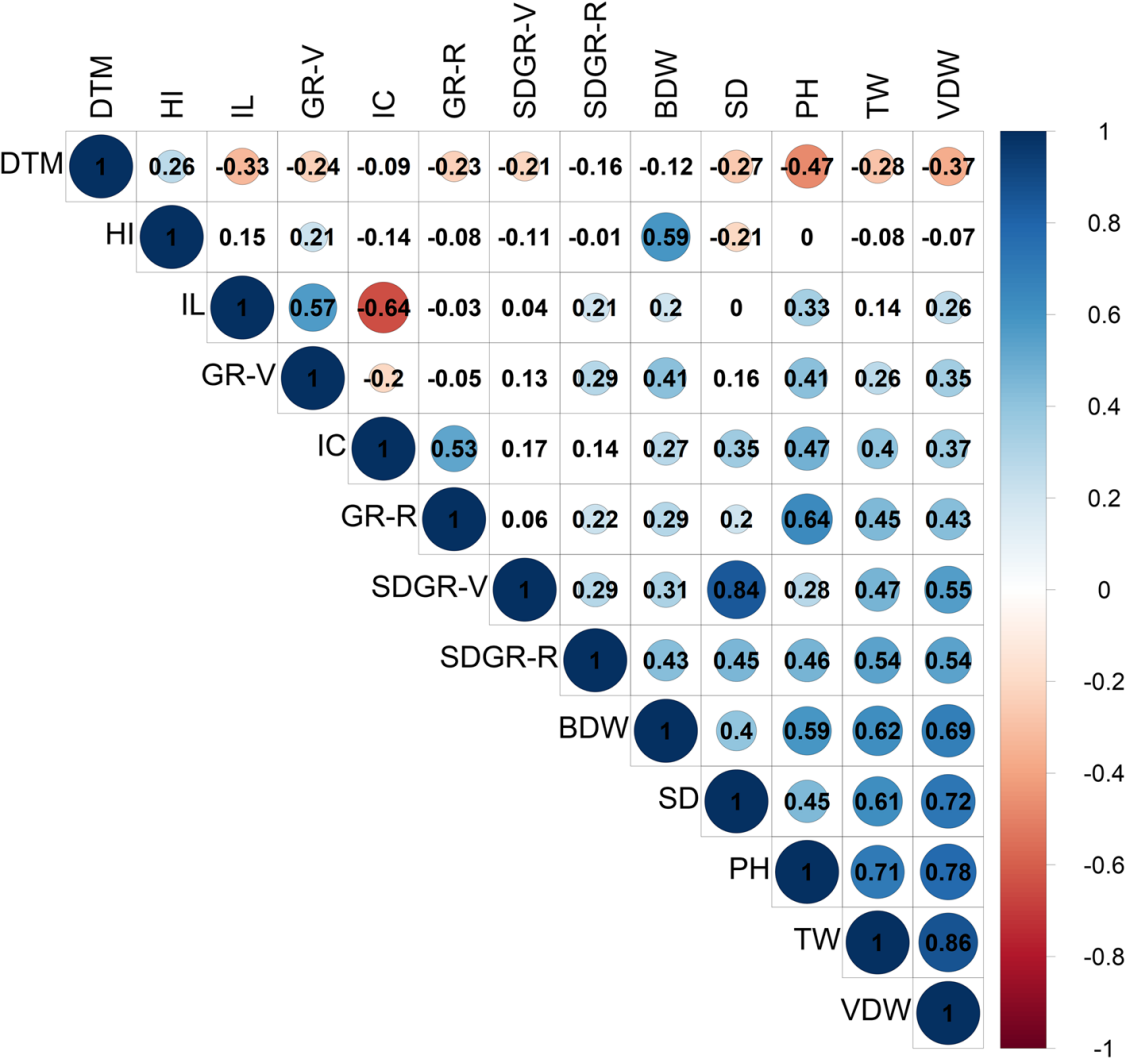
Correlation coefficient plot for 13 physiological parameters generated using the *corrplot* package [54]. Values and colours indicate correlation strength and direction respectively. Values marked with a background colour are statistically significant (*p *< 0.05). Abbreviations: Days to maturation (DTM), Harvest Index (HI), Internodes length (IL), plant height Growth Rate during Vegetative stage (GR-V), Internodes Count (IC), plant height Growth Rate during Reproductive stage (GR-R), Stem Diameter Growth Rate during Vegetative stage (SDGR-V), Stem Diameter Growth Rate during Reproductive stage (SDGR-R), Bud Dry Weight (BDW), Stem Diameter (SD), Plant Height (PH), Trimmed Waste (TW), Vegetative Dry Weight (VDW)

The trait DTM is negatively correlated with all presented growth-related traits (Fig. 3B). In most cases (9 of 12), this correlation was found to be statistically significant with the strongest coefficient found between DTM and PH (r = -0.47), while no significant correlations were observed between DTM to either BDW or SDGR-R (Fig. 4). A close association between SDGR-R, PH and HI was observed with plant productivity (BDW, Fig. 3B and C). This association is statistically validated by the correlation matrix (Fig. 4 and Additional file 1: Table S1) with coefficient ratios of r = 0.43, *p *< 0.001; r = 0.59, *p *< 0.001; r = 0.59, *p *< 0.001 for BDW to SDGR-R, PH and HI, respectively. Moreover, a statistically significant moderate (positive) correlation was identified between BDW and the vegetative stage growth rate attributes; GR-V (r = 0.41, *p *< 0.001) and stem diameter growth rate during the vegetative stage (SDGR-V, r = 0.31, *p *< 0.001, Fig. 4 and Additional file 1: Table S1).

### Broad-sense heritability

Estimated broad-sense heritability (H^2^) for physiological and phenological traits ranged from 0.3–0.78 (Tables 1 and 2). The parameters stem diameter (SD), PH and DTM are characterized by higher H^2^ values while the yield component parameters (such as; BDW, trimmed waste: TW, HI) present with H^2^ values in the lower range. With regards to weekly estimations of plant height and stem diameter (Table 2), higher H^2^ values (0.52 – 0.78) were observed relative to all of the reported H^2^ estimates presented in Table 1 (0.3 – 0.53). As the trial progressed, an overall reduction in H^2^ was observed across the weekly plant height trait (ranges from 0.78 to 0.52), while weekly H^2^ values for stem diameter remained almost constant across the trial (ranges between 0.52—0.56). Furthermore, the short daylight induction and the transition into the reproductive stage did not seem to impact the variability of H^2^ indices.

**Table 1:** Broad-sense heritability (H^2^) for physiological and phenological traits

Parameter	H^2^
Bud Dry Weight	0.33
Days to Maturation	0.49
Stem Diameter	0.53
Plant Height	0.52
Harvest Index	0.30
Internode Count	0.47
Trimmed Waste	0.30
Vegetative Dry Weight	0.44
Internode Length	0.35

**Table 2:** Weekly based broad-sense heritability (H^2^) for plant’s development during the vegetative and reproductive stages

Parameter	Growth stage	Week from planting	H^2^
Plant Height	Vegetative	2	0.78
		3	0.70
		4	0.56
	Reproductive	5	0.53
		6	0.55
		7	0.58
		8	0.57
		9	0.52
Stem Diameter	Vegetative	2	0.54
		3	0.56
		4	0.56
	Reproductive	5	0.52
		6	0.53
		7	0.52
		8	0.53

### Multiple regression and prediction equation

Multiple regression model was run to predict BDW from GR-V, SDGR-V and the plant height on the second week following planting (PH-W_2_). All assumptions regarding linearity (assessed by partial regression plots and a plot of studentized residuals against the predicted), independence of residuals (Durbin-Watson statistic of 2.008), homoscedasticity (assessed by visual inspection of a plot of studentized residuals versus unstandardized predicted values), multicollinearity (tolerance values > 1) and normality (assessed by a Q-Q Plot) have been met.

The multiple regression model significantly predicted BDW, F(3, 117) = 21.42, *p *< 0.001, adjusted R^2^ = 0.34. All three independent variables added statistical significance to the model prediction (*p *< 0.05). Regression coefficients and standard errors can be found in Table 3.

**Table 3:** Multiple regression results for total floral bud yield

BDW	B	95% CI for B		SE B	β	R^2^	ΔR^2^
		**LL**	**UL**				
**Model**						0.36	0.34^c^
**Constant**	- 77.33^c^	-113.81	- 40.84				
**GR-V**	4.33^c^	2.46	6.21	0.95	0.345^c^		
**SDGR-V**	5.94^a^	0.21	11.67	2.89	0.16^a^		
**PH- W1**	119.88^c^	67.82	171.94	26.29	0.357^c^		

*B* Unstandardized regression coefficient, *CI* Confidence interval, *LL* Lower limit, *UL* Upper limit, *SE* *B* Standard error of the coefficient, β standardized coefficient, R^2^ coefficient determination, ΔR^2^ Adjusted R^2^, *GR-V* Growth rate during the vegetative stage, *SDGR- V* Stem diameter growth ratio during the vegetative stage, *PH- W1* Plant height in the first measurement (2 weeks from planting)

^a^ Significant at the 0.05 probability level; ^b^ significant at the 0.01 probability level; ^c^ significant at the 0.001 probability level

From the coefficient values presented (Table 3), an increase of 1 cm*week^−1^ in GR-V, 1 mm*week^−1^ in SDGR-V or 1 m in PH-W_2_ are associated with an increase of 4.33, 5.94 and 119.88_gr_ of BDW, respectively. Based on this model, a predicted value of BDW can be estimated by the following equation:

1 $${DBW}_{gr}= -77.33 + 4.33{(GR-V)}_{\frac{cm}{week}}+5.94{(SDGR-V)}_{\frac{mm}{week}} +119.88 {(PH-{W}_{2})}_{m}$$

**Fig. 5:**
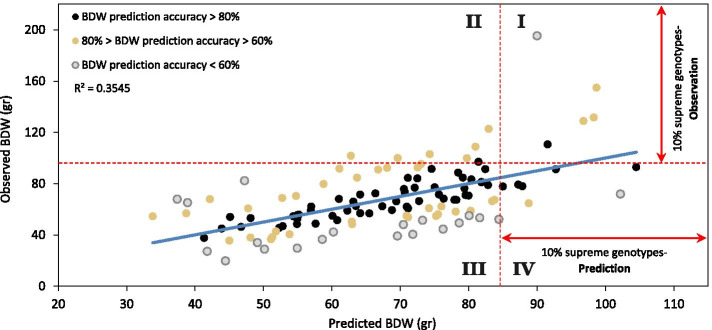
Scatterplot for observed versus predicted Bud Dry Weight (BDW). Predicted values are derived from the multiple regression prediction equation (Eq. 1). Colours represent prediction accuracy; black colouring consist of 59 genotypes and mark the prediction within 80%-100% accuracy, brown colouring consist of 41 genotypes and mark the prediction within 60%—80% accuracy and grey colouring consist of 21 genotypes and mark the prediction of less than 60% accuracy. Red broken lines mark the BDW value which defined the top 10% observed and predicted genotypes (out of the 121 examined genotypes)

In order to evaluate the accuracy of the prediction equation, a scatterplot of predicted vs observed BDW values was generated (Fig. 5). For each genotype, the deviation between the predicted and the observed values was calculated (predicted value divided by observed value) and from the ratio, genotypes were classified into groups, reflecting levels of prediction accuracy (demonstrated by the different colours in Fig. 5).

With the purpose of assessing the prediction equation for its potential breeding application and early selection of desired genotypes, comparisons between the top 10% performing predicted and observed lines (12 genotypes) were made (selection thresholds are marked by red broken lines in Fig. 5).

For 5 of the 12 top-performing lines, the prediction was accurate (Fig. 5, section I) while the 7 remaining genotypes were observed as false negatives with a prediction value below the selection threshold (Fig. 5, section II). Following this, 7 genotypes were identified as false positives with a prediction value above the selection threshold and an observed value below the selection threshold (Fig. 5, section IV). One hundred and two genotypes that were observed below the selection threshold (Fig. 5 section III) were predicted by the equation to be in this category (93.5% accuracy).

The examination of different selection thresholds suggests that by applying more moderated selection pressure (e.g. selecting the top 15%, 20% or 33% of the genotypes), the probability to unintentionally cull desired genotypes is reduced but at the cost of selecting more lines with mediocre performance (Additional file 2: Figure S1).

## Discussion

The examined plant population in the current study was based on diverse drug-type medicinal cannabis germplasm and its cannabinoid profile was dominated by high-THC and blended THC:CBD lines. As all of the strains under evaluation originated from legal medicinal cannabis companies, the findings of the current study indicate that despite the significant recreational breeding for high THC plants that took place in recent decades [33–35, 38] and a reduction in the genetic diversity of modern strain’s cannabinoid profile [28, 55], a vast phenotypic diversity across physiological and phenological traits still remains (Fig. 1). To extend these findings to High-CBD:low-THC genotypes that could have undergone different selection pressures, future studies are needed.

The establishment of the fast-growing medicinal cannabis industry requires scientifically based protocols for the optimization of yield quality and productivity under various growth conditions. However, to date, scarce research or globally-accepted protocols are available to support advanced cultivation of medicinal cannabis [56, 57]. Thus, understanding the physiological and phenological factors that determine the plant’s phenotypic expression can improve the adaptation of cannabis lines to different cultivation environments and optimize yield production and quality by tailoring strain-specific growth protocols [47, 49]. As space in cultivation facilities is often restricted (height-wise) and robust cultivars frequently require external support to prevent tilting or breakage through the weight of resinous inflorescences, one of the study’s aims was to characterize plant height and stem diameter growth patterns in order to understand their developmental mechanisms in relationship to floral bud yield. In general, plant growth is proposed to cease three weeks after the short daylight induction [4, 16] and although most examined genotypes in the current trial met this rule, our examination of the growth pattern trends identified that the final plant height and stem diameter state is determined by several factors that varied across genotypes (as demonstrated in Fig. 2). These factors include (1) growth rate during the reproductive and vegetative stages; (2) plant size on planting day; (3) the duration which passes before the impact of the daylight change is observed; (4) the type of growth deceleration response (deceleration intensity) and (5) the overall period between short daylight induction and complete growth cessation. Interestingly, a comparison between plant height and stem diameter growth patterns suggests that the transition into the reproductive stage has a somewhat limited effect on stem diameter growth rate while plant height growth rate was more susceptible to this change. Since the duration of each cultivation stage can be manipulated to enhance yield productivity [2, 58], quantifying the contribution of each of these factors to the phenotypic expression can provide better tools to generate optimal growing protocols and adapt cultivation to diverse growth conditions. In the current study, a single vegetative growth period was applied across all genotypes, which may have implications over the plant’s productivity. However, the conditions applied in this study are representative of common commercial protocols.

The majority of medicinal cannabis plant genetic material in use by the industry originated from clandestine breeding attempts [2] with a total estimation of 2,492 unique drug-type cannabis strains available globally as of June 2019 [38]. However, the main guidelines which directed the classification of genotypes to strain groups were subjected to the plant’s inebriant potency, morphology and its vernacular ancestry affiliation while the plant’s cannabinoid content was not of a major consideration [32, 37, 38]. As a result, the cannabinoid profile of plants which are associated with the same strain name is often inconsistent [32, 37, 59]. In the current trial, the variation of physiological and phenological traits across genotypes showed as much diversity within as between ‘cultivar’ (Fig. 3A), therefore, the vernacular names and classification is also inconsistent for validating morphological characteristics. This empirical evidence endorses the Hazekamp et al., (2016) [32] observation which noted that plants with the same strain name often look dissimilar. These findings strongly support and underpin the necessity of a genetically based classification system for a reliable characterization of cannabis genotypes for medicinal application [25].

Additionally, a broad observation over the traits’ distribution (as presented in Fig. 3A and B) suggest that precocious genotypes (low DTM) develop fast (high growth rate during the reproductive stage: GR-R and GR-V), but at the same time, no clear correlation was detected between DTM and BDW indices (Fig. 3C). Therefore, it is suggested that it will be relatively easy to breed for precocious varieties which are also high in yield. This assertion can be also corroborated by the non-significant correlation between DTM and BDW (r = -0.12, Fig. 4). Nevertheless, although plants precocity does not dependably predict productivity, the final PH can provide a reliable indication for BDW (r = 0.59) and DTM (r = -0.47). These findings suggest that higher plants often mature early and are more productive and therefore, it is important to better understand the factors which determine the final PH. Accordingly, associations between GR factors and the final PH demonstrate that a persistent and vigorous growth rate during the first 3–4 weeks of the reproductive stage (GR-R) has a strong significant correlation with the final PH (r = 0.64) compared with the GR during the vegetative stage (GR-V, r = 0.41), but a direct correlation between GR-R and BDW was found to be relatively weak (r = 0.29). Furthermore, the final PH can be estimated also by multiplying internodes count and their average length (IC x IL). However, the correlation coefficient values suggested that IC can be a better predictor of final PH (r = 0.47) than IL (r = 0.33). As IC can be measured before growth termination, it can help estimate the final plant height earlier in the growth cycle and support breeding initiatives which aim to increase productivity through selection for high stature plants.

A positive correlation was observed between SD and BDW (r = 0.4). This association indicates that breeding for thick stems might also assist in increasing yield production (and vice versa) and can help facilitate stable plant architecture that may replace or reduce the need for artificial plant supports. According to SDGR and in contrast to plant height GR, it is evident that the development during the vegetative stage (SDGR-V) has a stronger correlation with the final SD (r = 0.84) than SDGR-R (r = 0.45). For breeding purposes, these findings suggest that in order to select plants with thicker stems, it will be adequate to evaluate the SDGR at a vegetative stage alone.

Examination of the variation within genotypes across different sections of the CE facility can be attributed to the increased radiation and airflow over these areas (Additional file 3: Figures S1-S24). To address this limitation, it is suggested that future trial design will aim to reduce edge terrains, for example, by conducting experiments in a large square shape. Since this experiment was performed in a CE facility and spatial adjustments were applied in order to minimize the impact of abnormal environmental position effects, the broad-sense heritability (H^2^) values depicted in Tables 1 and 2 are reliably representing the degree of phenotypic variation (V_P_) that can be attributed to genetic factors (V_G_). Similar to the heritability values typifying hemp phenology [60, 61], the H^2^ of DTM (Table 1) is characterized by a relatively high value (0.49) and therefore, it is expected to enable a strong response to selection for this trait. Broad sense heritability values for the final PH were also found to be relatively high (0.52), but interestingly, the heritability of weekly growth patterns decreases during the vegetative growth phase before stabilising. This phenomenon can be ascribed to an increase in V_P_ as the vegetative stage progresses due to an accumulative effect of environmental factors influencing plants performance over time. In contrast, the weekly H^2^ for SD remained relatively constant during both cultivation stages (0.52–0.56) and it’s therefore suggested that a similar partition between genetic and environmental factors is determining SD phenotypic expression throughout the whole cultivation cycle. However, a comparison between previous narrow-sense heritability (h^2^) measurement for SD in hemp (0.22, [61]) and the current H^2^ ratio (0.53) suggests that further examination of SD h^2^ in cannabis is required in order to better understand its response to selection.

Limited cultivation space for undertaking selection purposes is a major obstacle for generating advanced cultivars through breeding. In medical cannabis cultivation, space limitation is an even prominent issue, as its cultivation is often dictated by stringent regulations and procedures. Therefore, improving breeding efficacy by selecting desired genotypes without the completion of a full cultivation cycle can greatly help to generate superior cultivars and advance the medicinal cannabis industry. The presented prediction equation for forecasting BDW suggests a reliable method for selection of desired genotypes according to physiological parameters which were recorded during the vegetative growth phase. As demonstrated in Fig. 5, this equation can indicate the most prolific plants (top 4 genotypes) and can also be used to cull undesired genotypes (102 of 121 under 90% selection pressure). Despite a number of prediction errors (false positives and negatives), this equation gives a possible solution for early-stage yield assessment of genetically diverse cannabis populations (drug types plants obtained from multiple resources and hemp background genotypes). However, it is likely that following several rounds of targeted selection, the plant population will become more homogeneous and the forecasting accuracy of this equation will improve up to a stage where the morphological differences between the parameters that drive the prediction equation have diminished. Therefore, this equation usage can be of most benefit when culling large sets of diverse germplasm, then selected elite cohorts will be screened via high-accuracy genomic selection methods. Although BDW is a crucial feature for commercial cultivars, combining and prioritizing other physiological and phenological traits together with plants' productivity should be considered in order to adjust plants morphology to a range of cultivation conditions and needs. Moreover, a significant part of the cannabis strains characterization should be dedicated to the plant secondary metabolites (cannabinoids and terpenes) which are essential for a scientifically based strain classification. For tailoring strain-specific cultivation techniques that can facilitate the standardization of secondary metabolites biosynthesis, a better understanding of the factors that determine the chemotypic variation between floral buds within the same plant is required [47]. Combining the physiological and phenological data provided in the current study together with the plant's cannabinoid profile can significantly advance the medicinal cannabis industry [61] and facilitate the commercialization of registered cannabis remedies. Future studies that will associate genome-wide variation with a comprehensive inventory of phenotypic traits will allow precise and accelerated breeding through genomic selection approaches.

## Conclusion

The findings presented in the current study suggest that despite the reduction in cannabinoid genetic diversity in recent decades, a vast phenotypic diversity across physiological and phenological traits still remains. Through breeding, this diversity can facilitate the generation of scientifically based genotypes that can be adapted to various growth conditions. A positive association was observed between inflorescences productivity, plant height and growth rate indicating that breeding for vigorous and fast-growing plants is likely to increase floral bud yield. In addition, 5 parameters have been identified to successfully determine the final profile of stem diameter and plant height. To better utilize the reproductive potential of each strain through cultivation adjustments, it is suggested that the characterization of medicinal cannabis cultivar should include its unique growth pattern features. Furthermore, a prediction equation for forecasting inflorescence yield was generated to facilitate the selection of desired genotypes without the completion of a full cultivation cycle. Together with the physiological and phenological characterization presented in the current manuscript, this equation provides breeding tools to advance the medicinal cannabis industry by improving breeding efficacy and generating scientifically based elite cultivars.

## Methods

### Experimental design

All seeds were legally imported from Canada or generated by project activities and all the work undertaken was performed under Medicinal Cannabis Research Licence (RL011/18) and Permits (RL01118P6 and RLO1118P3) issued by the Department of Health (DoH), Office of Drug Control (ODC), Australia.

**Fig. 6:**
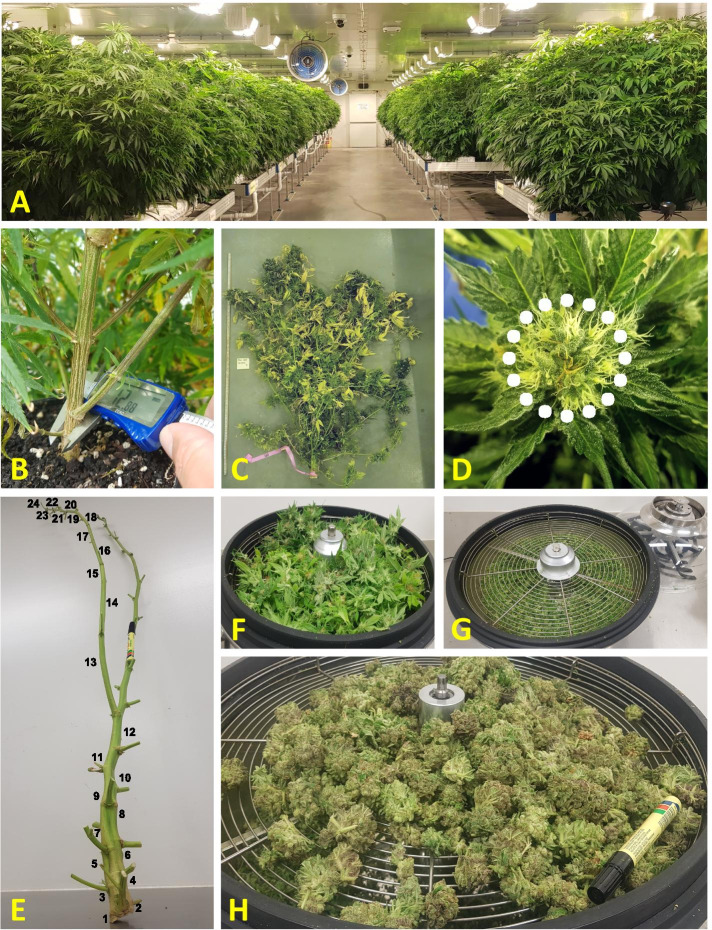
**A** Plant development in the controlled environment facility, **B** Stem diameter measurements, **C** Stature measurements, **D** Styles colour change during maturation, **E** Internodes count, **F** Raw material in leaf trimmer, **G** Trimmed by-product, **H** Processed buds

A diverse population of 121 genotypes, each of which generated from a genetically unique single seed, was maintained under CE conditions (Fig. 6A). Prior to the study, the phenotypic variation between clones of single genotypes was estimated and an optimal level of clonal replication was determined to be 4–5 (Additional file 4: Figures S1 and S2).

For all examined lines, an assessment of the major cannabinoids profile was performed by the B1080/1192 DNA marker [62] to characterize high THC and blended THC:CBD lines. Ninety-nine of the examined genotypes were derived from ‘cultivars’ of medicinal cannabis, however, this classification is more of a popular description rather than a scientifically based categorization. Within these ‘cultivar’ groups, according to the DNA marker results, 71 lines were classified with a high-THC:low-CBD ratio and 28 lines were classified with a ratio of blended THC:CBD. These results were in agreement with the suppliers’ declaration regarding the cannabinoid profile of the provided plant material. The remaining 22 genotypes have been developed by Agriculture Victoria Research as hybrids between lines with a diverse THC:CBD ratio. Within these accessions, 4 crosses were classified with a ratio of high-THC:low-CBD and 18 crosses were classified with a ratio of blended THC:CBD.

Stock (mother) plants for each genotype were maintained vegetatively and used to generate 10 clonal cuttings (10.5 ± 0.5 cm) each of which had rooting induced from hormones (Growth Technologies, Clonex, 3 g/L IBA gel, Perth, Australia) and planting took place in coconut coir propagation plugs (Jiffy-7C®, 50 mm, Zwijndrecht, Netherlands). Plant establishment took place in a CE nursery under long daylight regime (16 h/8 h) which provided by fluorescent tubes (Philips TL-D Reflex 58 W/840, Amsterdam, Netherland) delivering light intensity (PPFD) of 360 (μmol m^−2^ s^−1^) when measured 35 cm below the light source. The relative humidity (RH) was set to 55% and the temperature was fixed to 24/18 °C (day/night). Thirty days later, 6–7 rooted cuttings at similar developmental stage (height and vigour) were selected from the ten clones and transplanted into large coir plugs (Jiffypots®, ø8 cm, Zwijndrecht, Netherlands). A further selection for standardisation of growth was then imposed and the most uniform 4–5 plants were selected for transplanting. A randomised incomplete block design was developed using the R package *blocksdesign* [63]. The trial consisted of 19 growth benches arranged as two columns (with 1 bench missing from the first column due to a physical obstacle). Each bench contained 28 plants arranged in two doubled rows and 14 columns (Additional file 5: Figure S1). Benches were assigned as blocks and further parameters were included in the trial design to ensure no duplication of entries across the global 20 room rows and 28 columns.

### Growth conditions

Genotypes were planted in coconut coir substrates (Cazna grow slabs, Sydney, Australia) over a rolling bench system in ebb-flow trays (Danish Hydro Trays 338*148 cm, Ringe, Denmark) and spaced 20 cm and 40 cm within and between rows respectively in a density of 4.3 (plants/m^2^). High-pressure sodium lights (Philips, MASTER GreenPower Xtra 1000 W EL/5X6CT, Amsterdam, Netherland) provided a light intensity (PPF) of 2150 μmol s^−1^ and used to maintain 18 h and 12 h photoperiodic regime for vegetative and reproductive stages respectively. Relative humidity was 60% and the temperature maintained at 20/17 °C (day/night) for the entire growth period. Plants were fertigated twice daily (650 ml/plant for each cycle) using a regulated drip irrigation system (Jain Octa-Bubbler™, 7.5L/h, Fresno, California, USA), applying 1% A&B fertilizer solution (THC™, coco A + B, Melbourne, Australia) with EC ratio of 2.1 dS/m and pH levels of 6–6.1. The plants' vegetative growth phase duration was 42 days (from initial transplanting date) after which the reproductive stage was initiated by transferring to a short-daylight (12 h) regime. Later in the season, wooden stakes were attached to plants (where required) to prevent stems from tilting. Pest management was implemented by beneficial arthropods (*Neoseiulus californicus* *,* *Neoseiulus cucumeris* *,* *Dalotia coriaria* *,* *Hypoaspis aculeifer* and *Phytoseiulus persimilis*) that were frequently used during the growing season.

### Phenology and physiology characterization

SD was recorded weekly for 7 weeks, using electronic vernier calliper (Kincrome, 6", Melbourne, Australia) and an average growth rate for each genotype was calculated for the vegetative (SDGR-V) and the reproductive (SDGR-R) growth phases. In order to maintain stable measurements, a shallow incision was made between the first 2 second-order branches to set a consistent calliper situs (Fig. 6B). PH was recorded weekly for 8 weeks (Fig. 6C) and the data was used to calculate the growth rate of each genotype during vegetative (GR-V) and reproductive (GR-R) stages.

Plant phenology was evaluated every 3 days and is specified as the duration period between the transition to short-day reproductive induction and the plant's maturity (Day to Maturation: DTM). Maturation was defined as the stage where inflorescence style’s colour changed into a shade of brown on 3 independent floral buds (Fig. 6D). Harvest was carried out selectively when ~ 70% of plants styles had turned brown. Once harvested, Internodes were counted (IC) along the leading (or the highest) branch and an average internode length (IL) was calculated (Fig. 6E). Vegetative (foliage leaves within inflorescence and stems) and inflorescence plants parts were separated, and bud material was purified using a leaf trimmer (Fig. 6F-H; Growlush® bowl trimmer, 19", Melbourne, Australia). Vegetative material and trimmed by-product were oven-dried (65 °C for 72 h) and bud material was dried at room temperature (25 °C, 20% humidity) before being placed in a freeze dryer (VirTis, GPFD, Gardiner, NY, USA) for complete dehydration. Vegetative dry weight (VDW), trimmed waste (TW) and bud dry weight (BDW) were recorded and harvest index (HI) was calculated accordingly.

### Statistical analysis and spatial adjustments

Data analysis was conducted using IBM SPSS Statistics for Windows, Version 26.0 (Armonk, NY: IBM Corp) and by R [64]. Physiological and environmental variation was assessed throughout the facility through the experimental design. Best linear unbiased predictors (BLUPs) for each trait were calculated using *ASReml-R* package [65]. A two-dimensional separable autoregressive spatial structure (AR1⊗AR1) was fitted as the base spatial model to adjust for common variation across neighbouring plants. Residuals were summarised within rows and columns as well as being visualised in R [64] as a position based heatmap using the *ggplot2* package [66] to identify any spatial trends across the trial. Global spatial trends were typically observed along columns, within the margins of the cultivated benches (Additional file 3: Figure S1A through to Figure S24A). These were adjusted where appropriate by fitting column as a random effect within the model (Additional file 3: Figure S1B through to Figure S24B).

Broad sense heritability, H^2^, was calculated from ASReml models for all recorded parameters as the degree of phenotypic variation (V_P_) that can be attributed to genetic factors (V_G_) as H^2^ = V_G_/V_P_.

Despite the experiment being only a single trial within a fully CE setting, the application of detailed protocols, spatial adjustments and optimized replicate number increased the reliability and validated the generated data.

## Supplementary Information

**Additional file 1: Table S1** Correlation coefficient and P-values demonstrating the association between physiological traits.

**Additional file 2: Figure S1** Prediction equation under various ratios of selection pressure. This figure includes a scatterplot for observed versus predicted Bud Dry Weight (BDW) under different ratios of selection pressure. Predicted values are derived from the multiple regression prediction equation (Equation 1, see manuscript). Dot point colours represent prediction accuracy; black colouring consists of 59 genotypes and marks the prediction within 80%-100% accuracy, brown colouring consists of 41 genotypes and marks the prediction within 60% - 80% accuracy and grey colouring consists of 21 genotypes and marks the prediction that is less than 60% accuracy. Red, green, blue and yellow broken lines indicate the BDW value which defined the top 10%, 15%, 20% and 33% observed and predicted genotypes, respectively. Section I signifies matches between predicted and observed high performing genotypes; Section II contains genotypes identified as false negatives by the prediction equation; Section III signifies matches between predicted and observed low performing genotypes and Section IV contains genotypes identified as false positives by the prediction equation.

**Additional file 3.**

**Additional file 4: **The determination of replicate quantities.

**Additional file 5: Figure S1 **Trial design and spatial arrangement. Black boxes represent rolling benches, red and blue lines represent room rows and room columns, respectively and green numbers indicate the first and last plot on each bench.

## Data Availability

The datasets used and/or analysed during the current study are available from the corresponding author on reasonable request.

## Abbreviations

CE: Controlled environment
RH: Relative humidity
SD: Final measurement of stem diameter
SDGR-V: Stem diameter growth rate during the vegetative stage
SDGR-R: Stem diameter growth rate during the reproductive stage
PH: Plant height on harvest day
GR-V: Height growth rate during the vegetative stage
GR-R: Height growth rate during the reproductive stage
DTM: Days to maturation
IC: Internodes count on the leading branch
IL: Average internode length
VDW: Vegetative dry weight
TW: Trimmed waste
BDW: Floral bud dry weight
HI: Harvest index
