# Supplementary material for: The characterization of key physiological traits of medicinal cannabis (Cannabis sativa L.) as a tool for precision breeding
Source: BMC Plant Biol. 2021 Jun 26;21:294. doi: 10.1186/s12870-021-03079-2 (PMC8235858; doi:10.1186/s12870-021-03079-2)
Supplement: Supplementary file 2 — Additional file 2: Figure S1 Prediction equation under various ratios of selection pressure. This figure includes a scatterplot for observed versus predicted Bud Dry Weight (BDW) under different ratios of selection pressure. Predicted values are derived from the multiple regression prediction equation (Equation 1, see manuscript). Dot point colours represent prediction accuracy; black colouring consists of 59 genotypes and marks the prediction within 80%-100% accuracy, brown colouring consists of 41 genotypes and marks the prediction within 60% - 80% accuracy and grey colouring consists of 21 genotypes and marks the prediction that is less than 60% accuracy. Red, green, blue and yellow broken lines indicate the BDW value which defined the top 10%, 15%, 20% and 33% observed and predicted genotypes, respectively. Section I signifies matches between predicted and observed high performing genotypes; Section II contains genotypes identified as false negatives by the prediction equation; Section III signifies matches between predicted and observed low performing genotypes and Section IV contains genotypes identified as false positives by the prediction equation. [file 12870_2021_3079_MOESM2_ESM.docx]

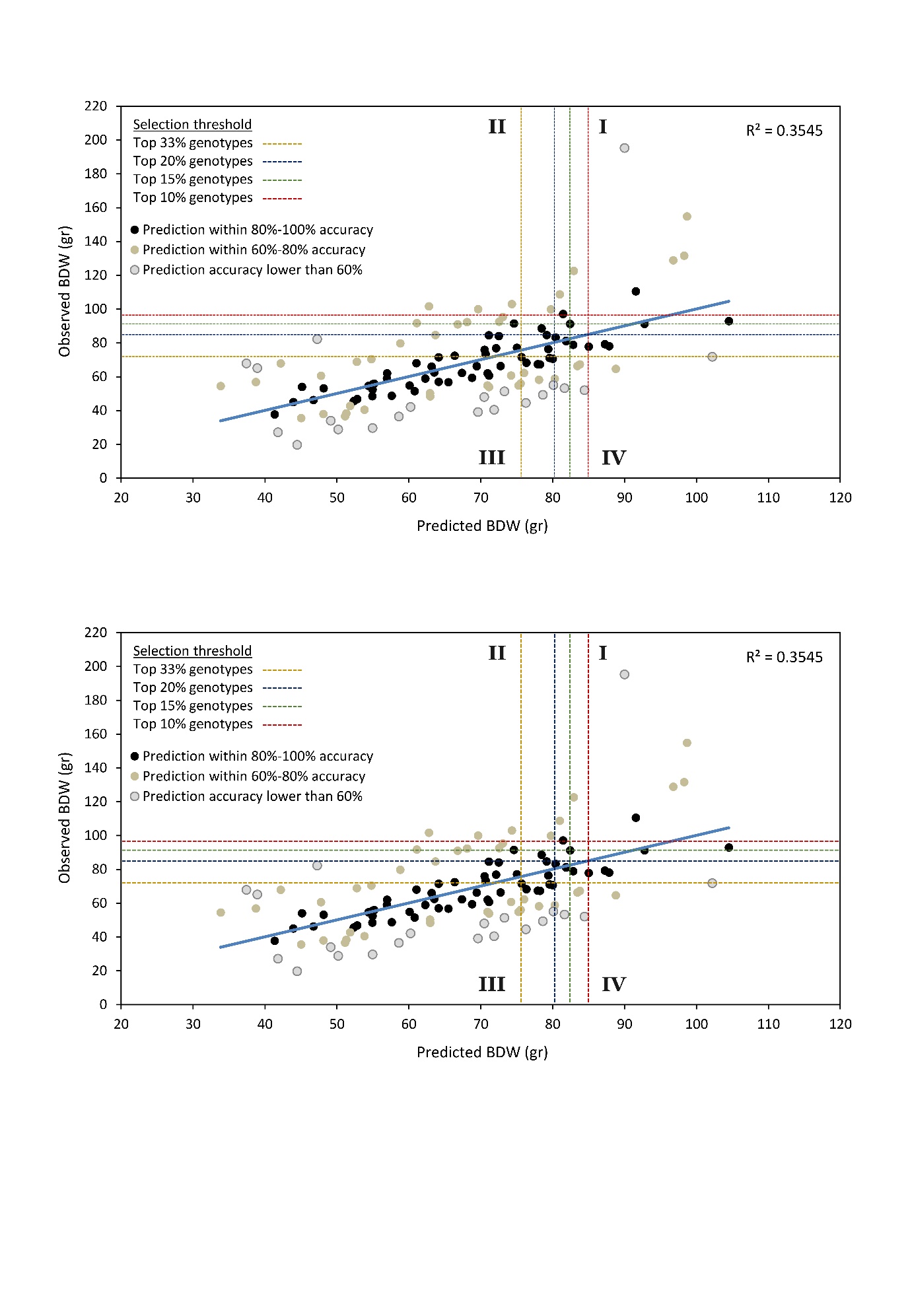


Additional File 2: Figure S1. Prediction equation under various ratios of selection pressure. This figure includes a scatterplot for observed versus predicted Bud Dry Weight (BDW) under different ratios of selection pressure. Predicted values are derived from the multiple regression prediction equation (Equation 1, see manuscript). Dot point colours represent prediction accuracy; black colouring consists of 59 genotypes and marks the prediction within 80%-100% accuracy, brown colouring consists of 41 genotypes and marks the prediction within 60% - 80% accuracy and grey colouring consists of 21 genotypes and marks the prediction that is less than 60% accuracy. Red, green, blue and yellow broken lines indicate the BDW value which defined the top 10%, 15%, 20% and 33% observed and predicted genotypes, respectively.

Section I signifies matches between predicted and observed high performing genotypes; Section II contains genotypes identified as false negatives by the prediction equation; Section III signifies matches between predicted and observed low performing genotypes and Section IV contains genotypes identified as false positives by the prediction equation.
