# Supplementary material for: The characterization of key physiological traits of medicinal cannabis (Cannabis sativa L.) as a tool for precision breeding
Source: BMC Plant Biol. 2021 Jun 26;21:294. doi: 10.1186/s12870-021-03079-2 (PMC8235858; doi:10.1186/s12870-021-03079-2)
Supplement: Supplementary file 3 — Additional file 3. [file 12870_2021_3079_MOESM3_ESM.pdf]

Days To Maturation (DTM)

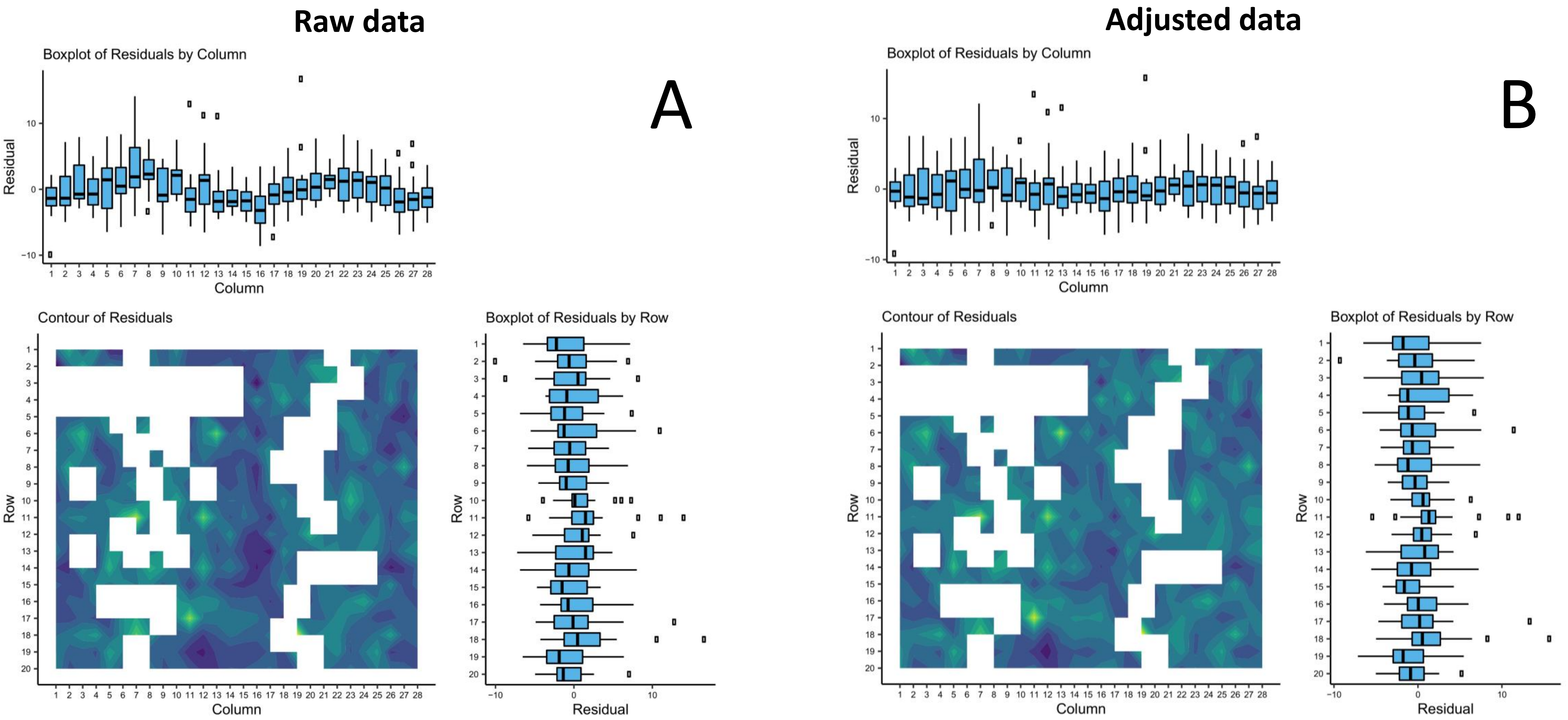

Additional File 3: Figure S1. A graphical representation of environmental variation within the controlled environment (CE) facility and the applied spatial adjustments. A: Raw data (prior to adjustments) which includes a heatmap for environmental variation across all positions within the CE facility as well as boxplots of the residuals to reflect the effect of environmental variations between rows and between columns for DTM. B: Adjusted data (after spatial adjustments) which includes a heatmap for environmental variation across all positions within the CE facility as well as boxplots of the residuals to reflect the effect of environmental variations between rows and between columns for DTM. Blank areas in the heatmaps represent missing data.

Harvest Index (HI)

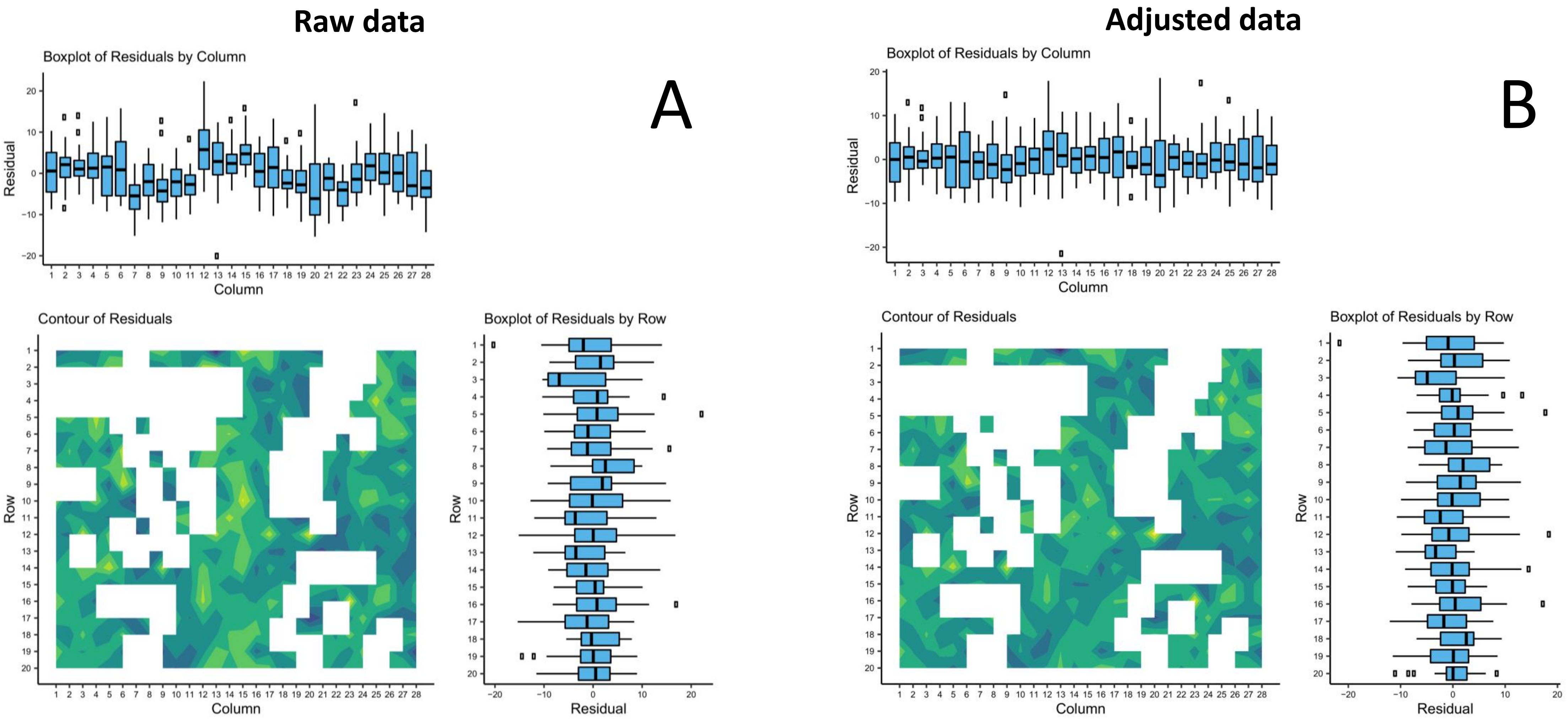

Additional File 3: Figure S2. A graphical representation of environmental variation within the controlled environment (CE) facility and the applied spatial adjustments. A: Raw data (prior to adjustments) which includes a heatmap for environmental variation across all positions within the CE facility as well as boxplots of the residuals to reflect the effect of environmental variations between rows and between columns for HI. B: Adjusted data (after spatial adjustments) which includes a heatmap for environmental variation across all positions within the CE facility as well as boxplots of the residuals to reflect the effect of environmental variations between rows and between columns for HI. Blank areas in the heatmaps represent missing data.

Plants Height On Harvest Day (PH)

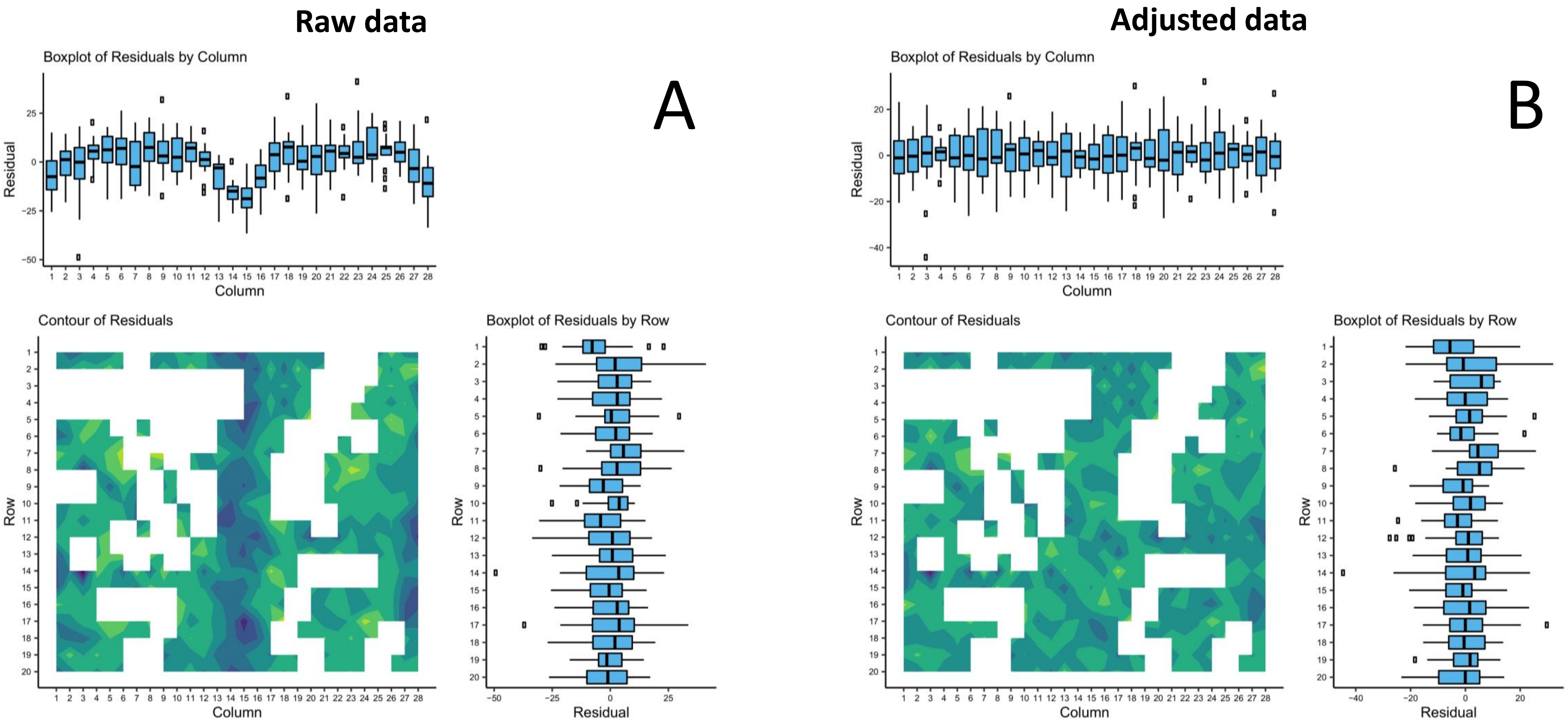

Additional File 3: Figure S3. A graphical representation of environmental variation within the controlled environment (CE) facility and the applied spatial adjustments. A: Raw data (prior to adjustments) which includes a heatmap for environmental variation across all positions within the CE facility as well as boxplots of the residuals to reflect the effect of environmental variations between rows and between columns for PH. B: Adjusted data (after spatial adjustments) which includes a heatmap for environmental variation across all positions within the CE facility as well as boxplots of the residuals to reflect the effect of environmental variations between rows and between columns for PH. Blank areas in the heatmaps represent missing data.

Stem Diameter (SD)

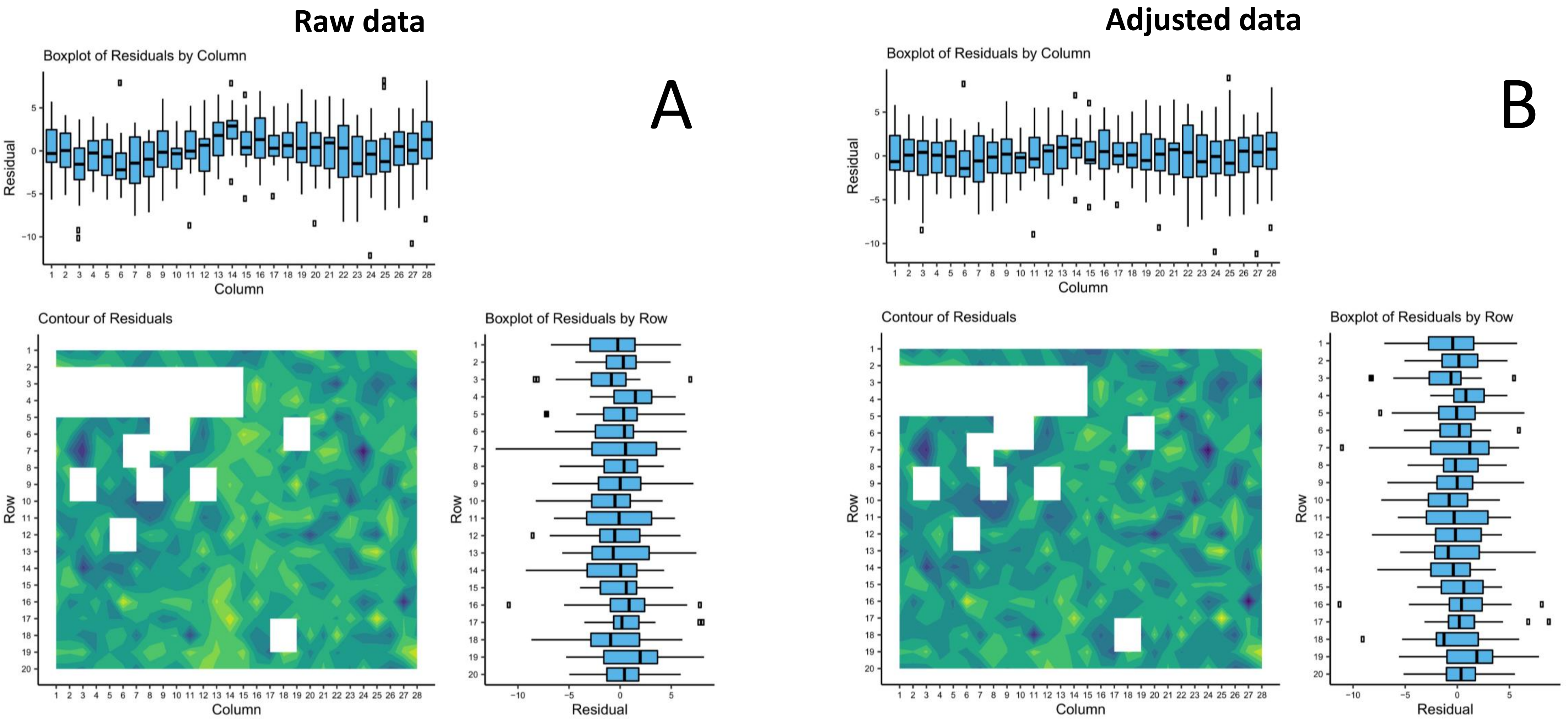

Additional File 3: Figure S4. A graphical representation of environmental variation within the controlled environment (CE) facility and the applied spatial adjustments. A: Raw data (prior to adjustments) which includes a heatmap for environmental variation across all positions within the CE facility as well as boxplots of the residuals to reflect the effect of environmental variations between rows and between columns for SD. B: Adjusted data (after spatial adjustments) which includes a heatmap for environmental variation across all positions within the CE facility as well as boxplots of the residuals to reflect the effect of environmental variations between rows and between columns for SD. Blank areas in the heatmaps represent missing data.

Bud Dry Weight (BDW)

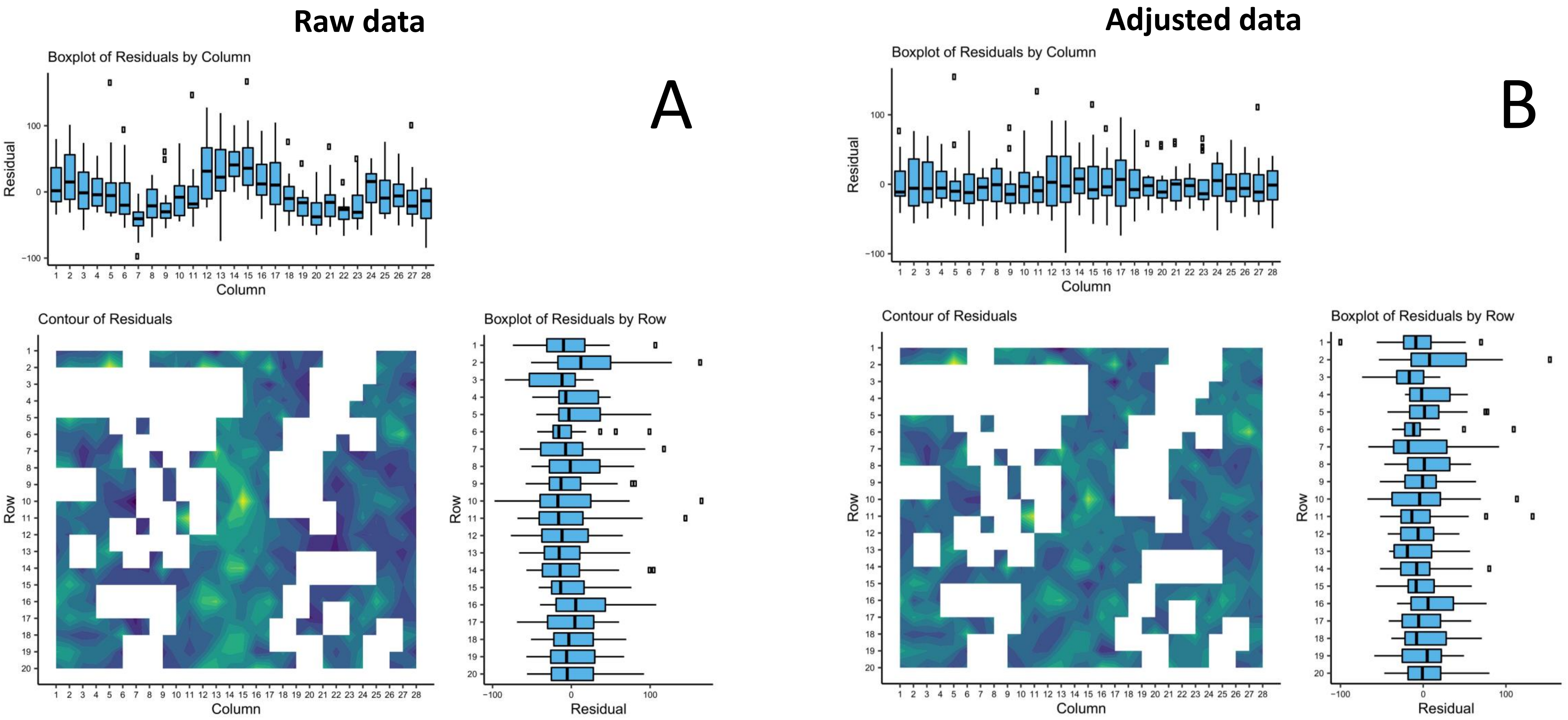

Additional File 3: Figure S5. A graphical representation of environmental variation within the controlled environment (CE) facility and the applied spatial adjustments. A: Raw data (prior to adjustments) which includes a heatmap for environmental variation across all positions within the CE facility as well as boxplots of the residuals to reflect the effect of environmental variations between rows and between columns for BDW. B: Adjusted data (after spatial adjustments) which includes a heatmap for environmental variation across all positions within the CE facility as well as boxplots of the residuals to reflect the effect of environmental variations between rows and between columns for BDW. Blank areas in the heatmaps represent missing data.

Internodes Count (IC)

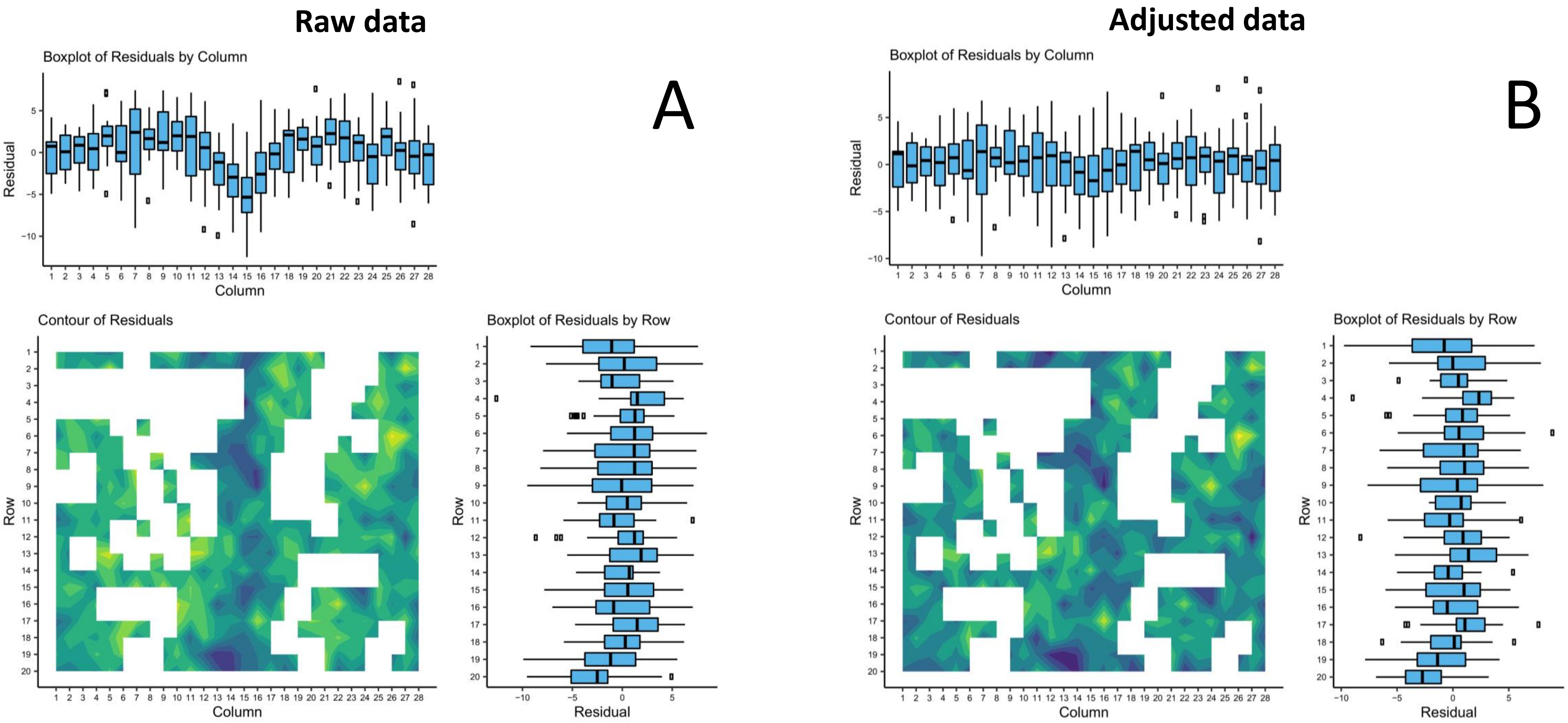

Additional File 3: Figure S6. A graphical representation of environmental variation within the controlled environment (CE) facility and the applied spatial adjustments. A: Raw data (prior to adjustments) which includes a heatmap for environmental variation across all positions within the CE facility as well as boxplots of the residuals to reflect the effect of environmental variations between rows and between columns for IC. B: Adjusted data (after spatial adjustments) which includes a heatmap for environmental variation across all positions within the CE facility as well as boxplots of the residuals to reflect the effect of environmental variations between rows and between columns for IC. Blank areas in the heatmaps represent missing data.

Trimmed Waste (TW)

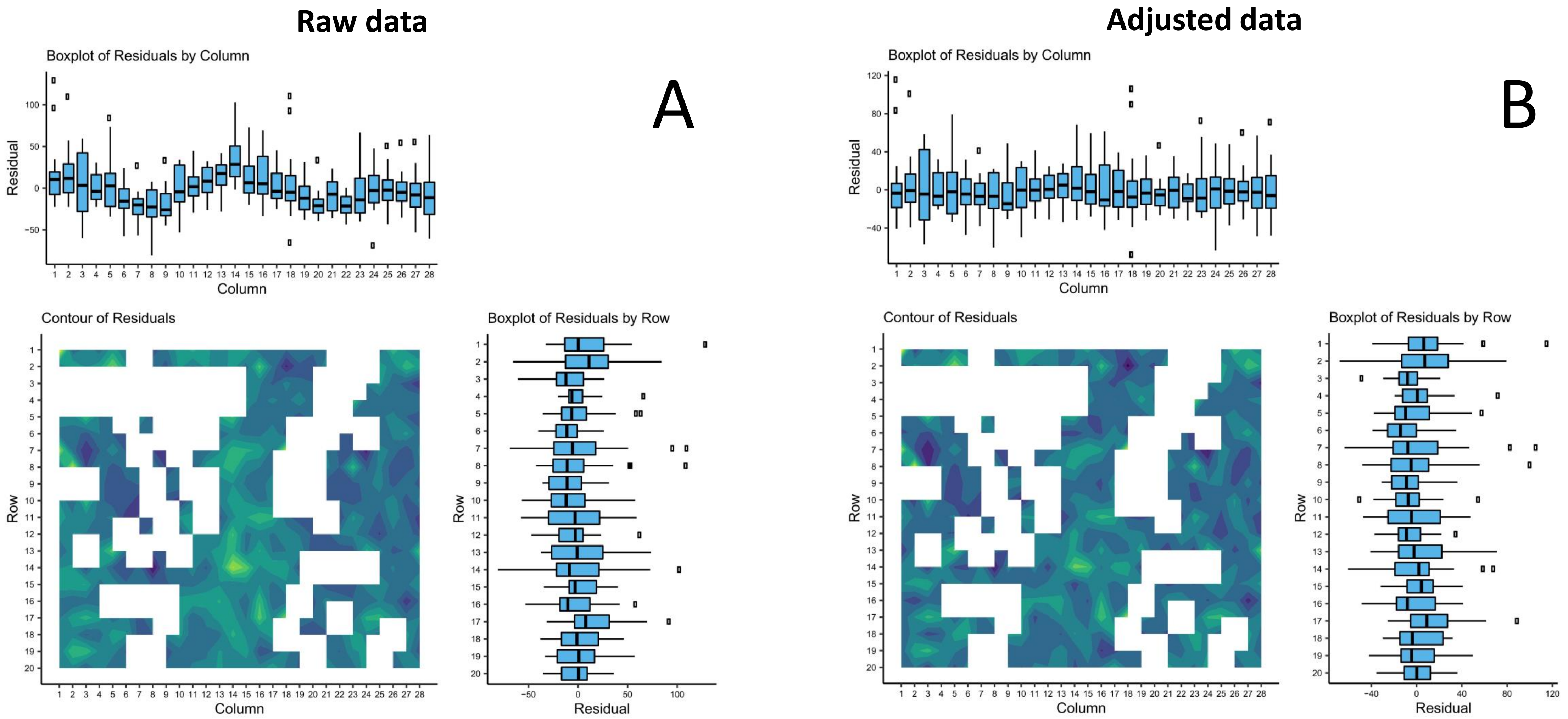

Additional File 3: Figure S7. A graphical representation of environmental variation within the controlled environment (CE) facility and the applied spatial adjustments. A: Raw data (prior to adjustments) which includes a heatmap for environmental variation across all positions within the CE facility as well as boxplots of the residuals to reflect the effect of environmental variations between rows and between columns for TW. B: Adjusted data (after spatial adjustments) which includes a heatmap for environmental variation across all positions within the CE facility as well as boxplots of the residuals to reflect the effect of environmental variations between rows and between columns for TW. Blank areas in the heatmaps represent missing data.

Vegetative Dry Weight (VDW)

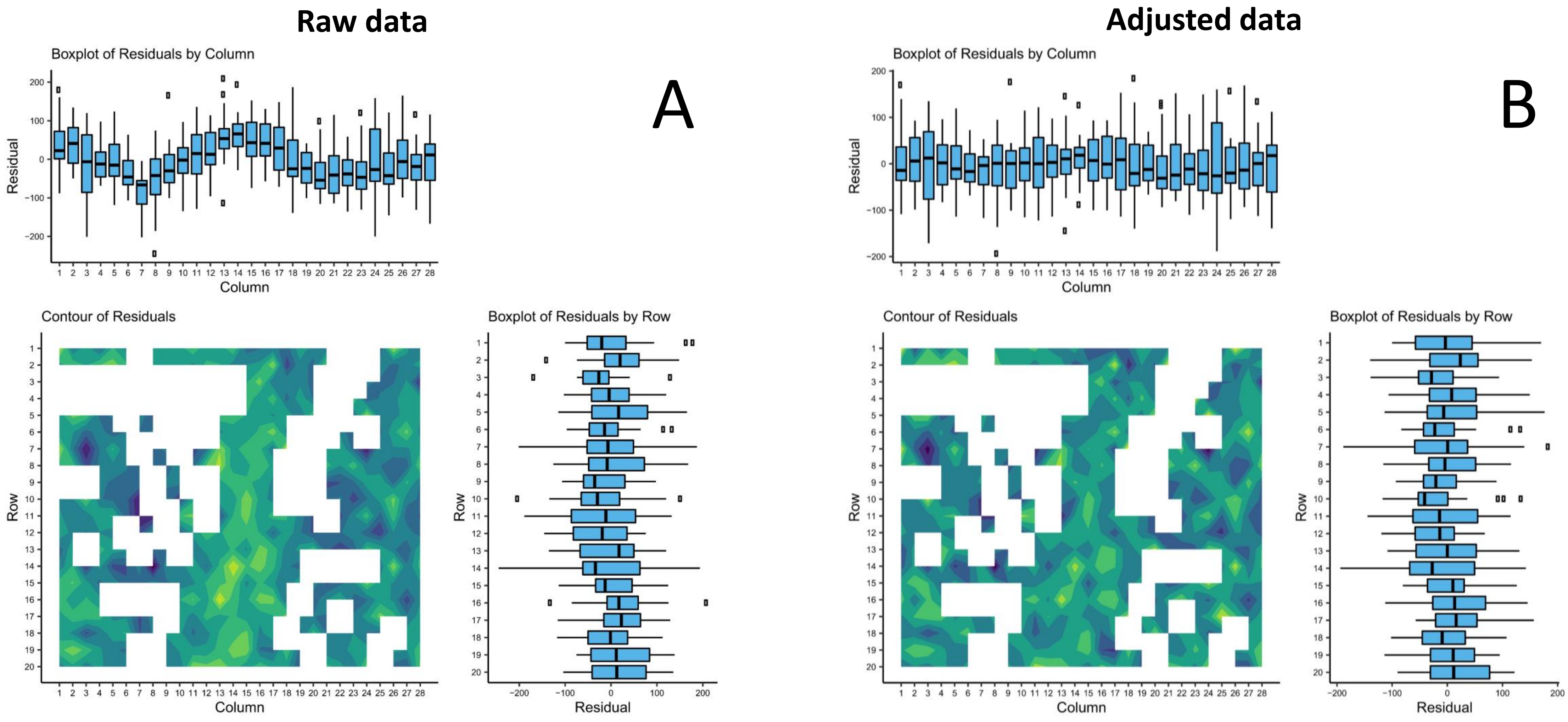

Additional File 3: Figure S8. A graphical representation of environmental variation within the controlled environment (CE) facility and the applied spatial adjustments. A: Raw data (prior to adjustments) which includes a heatmap for environmental variation across all positions within the CE facility as well as boxplots of the residuals to reflect the effect of environmental variations between rows and between columns for BDW. B: Adjusted data (after spatial adjustments) which includes a heatmap for environmental variation across all positions within the CE facility as well as boxplots of the residuals to reflect the effect of environmental variations between rows and between columns for BDW. Blank areas in the heatmaps represent missing data.

Internodes Length (IL)

A

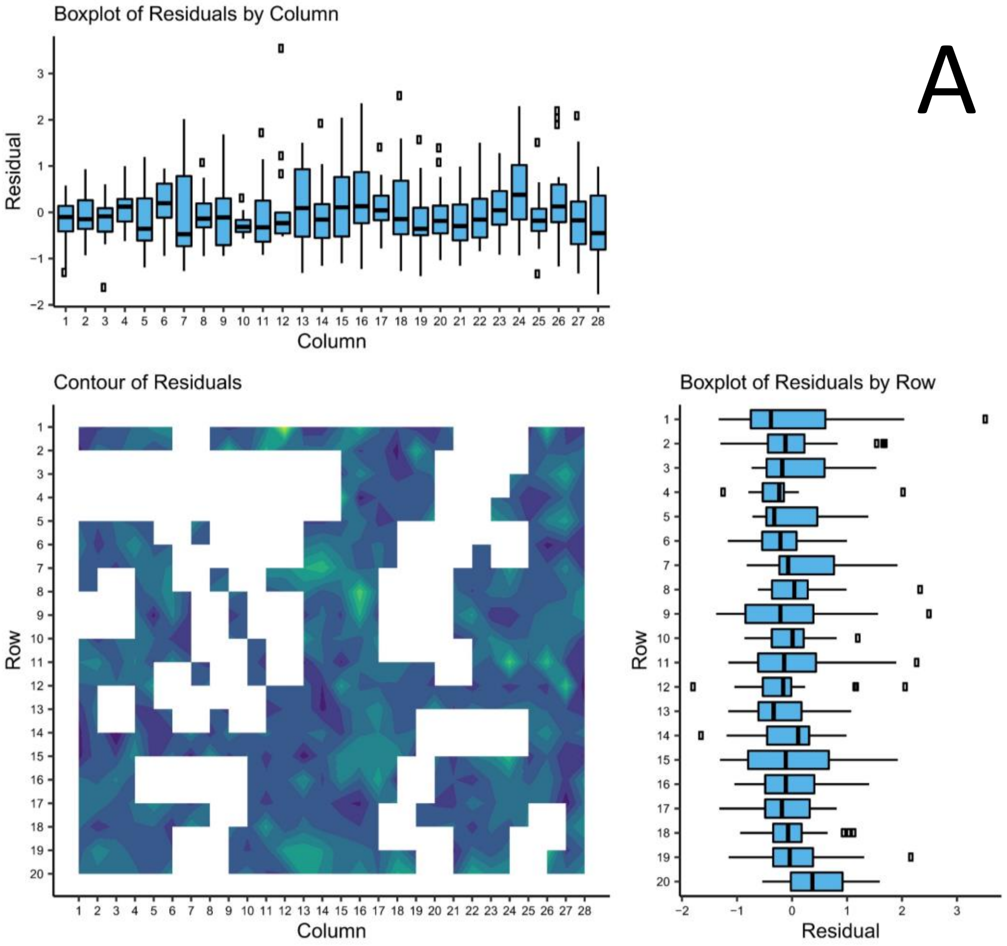

Additional File 3: Figure S9. A graphical representation of environmental variation within the controlled environment (CE) facility. Raw data is depicted via a heatmap for environmental variation across all positions within the CE facility as well as boxplots of the residuals to reflect the row and column position effect for IL. Blank areas in the heatmaps represent missing data. Spatial adjustments were not required and therefore were not conducted.

Plant Height- Week 2 (PH-W2)

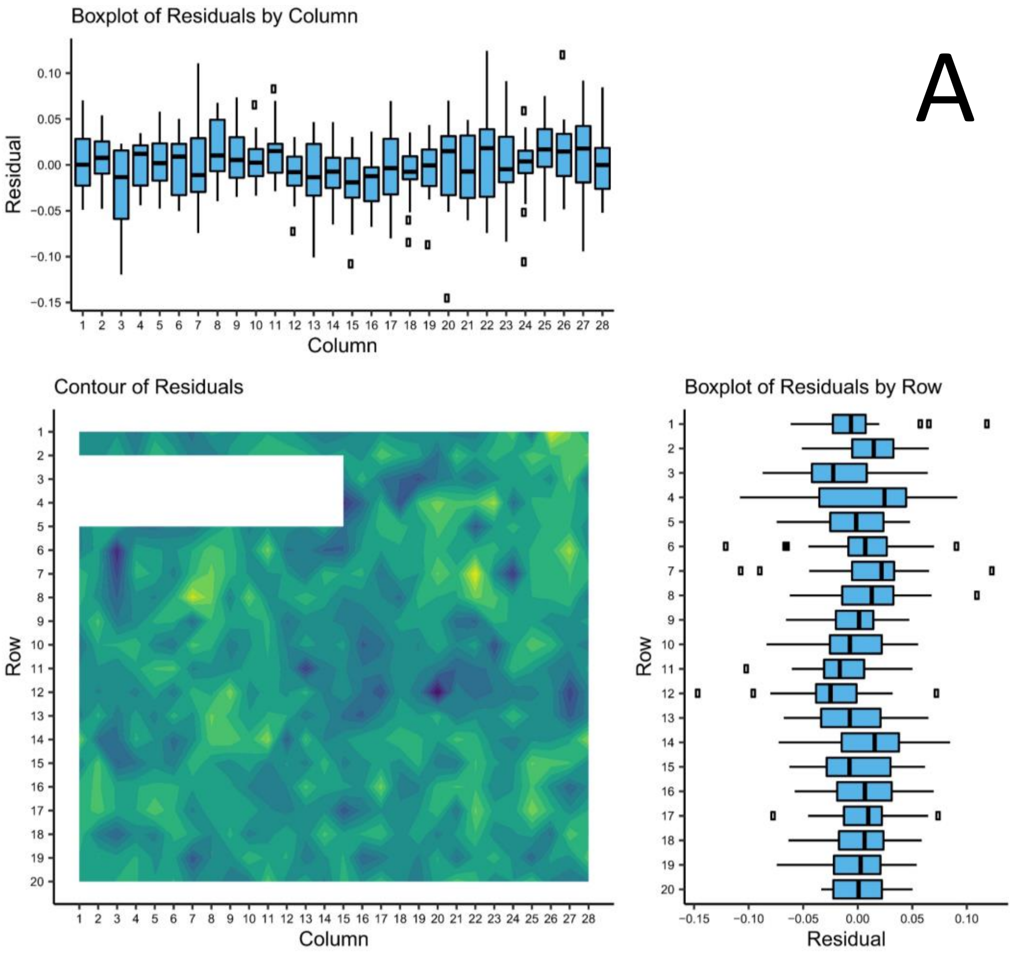

Additional File 3: Figure S10. A graphical representation of environmental variation within the controlled environment (CE) facility. Raw data is depicted via a heatmap for environmental variation across all positions within the CE facility as well as boxplots of the residuals to reflect the row and column position effect for PH-W2. Blank areas in the heatmaps represent missing data. Spatial adjustments were not required and therefore were not conducted.

Plant Height- Week 3 (PH-W3)

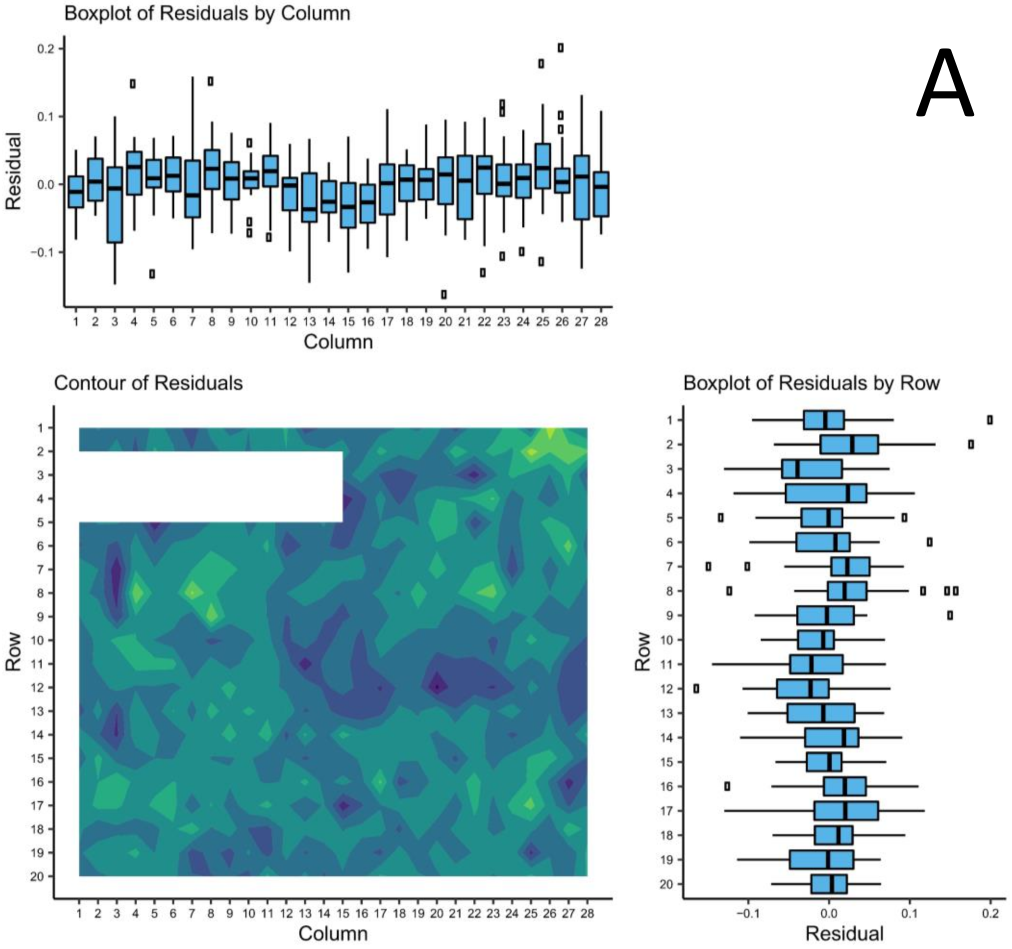

A

Additional File 3: Figure S11. A graphical representation of environmental variation within the controlled environment (CE) facility. Raw data is depicted via a heatmap for environmental variation across all positions within the CE facility as well as boxplots of the residuals to reflect the row and column position effect for PH-W3. Blank areas in the heatmaps represent missing data. Spatial adjustments were not required and therefore were not conducted.

Plant Height- Week 4 (PH-W4)

Raw data

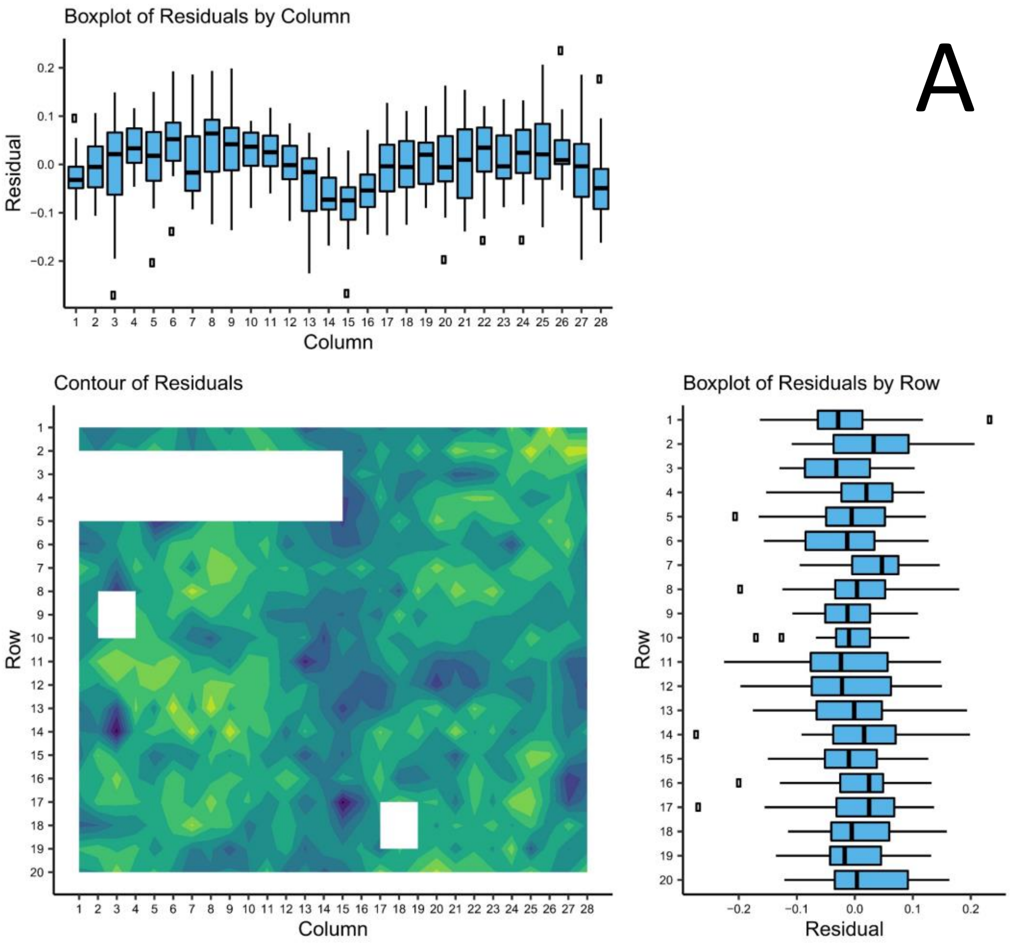

Adjusted data

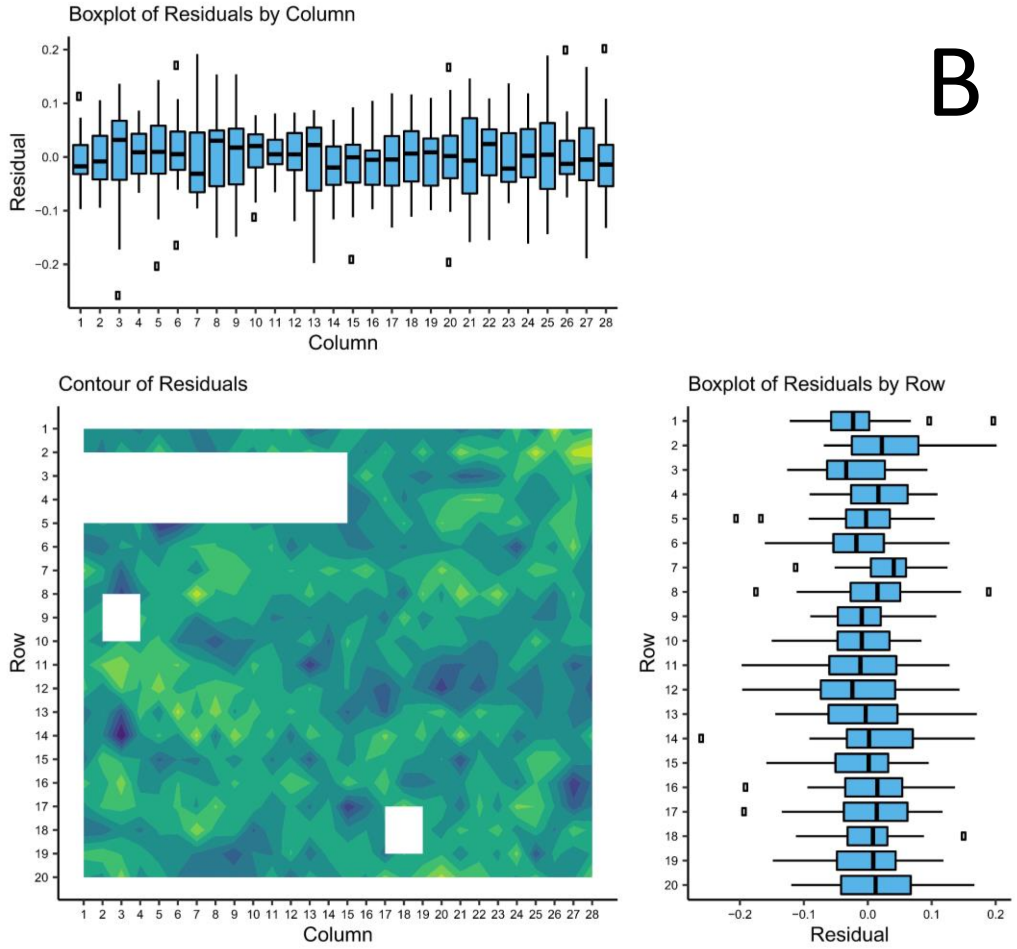

Additional File 3: Figure S12. A graphical representation of environmental variation within the controlled environment (CE) facility and the applied spatial adjustments. A: Raw data (prior to adjustments) which includes a heatmap for environmental variation across all positions within the CE facility as well as boxplots of the residuals to reflect the effect of environmental variations between rows and between columns for PH-W4. B: Adjusted data (after spatial adjustments) which includes a heatmap for environmental variation across all positions within the CE facility as well as boxplots of the residuals to reflect the effect of environmental variations between rows and between columns for PH-W4. Blank areas in the heatmaps represent missing data.

Plant Height- Week 5 (PH-W5)

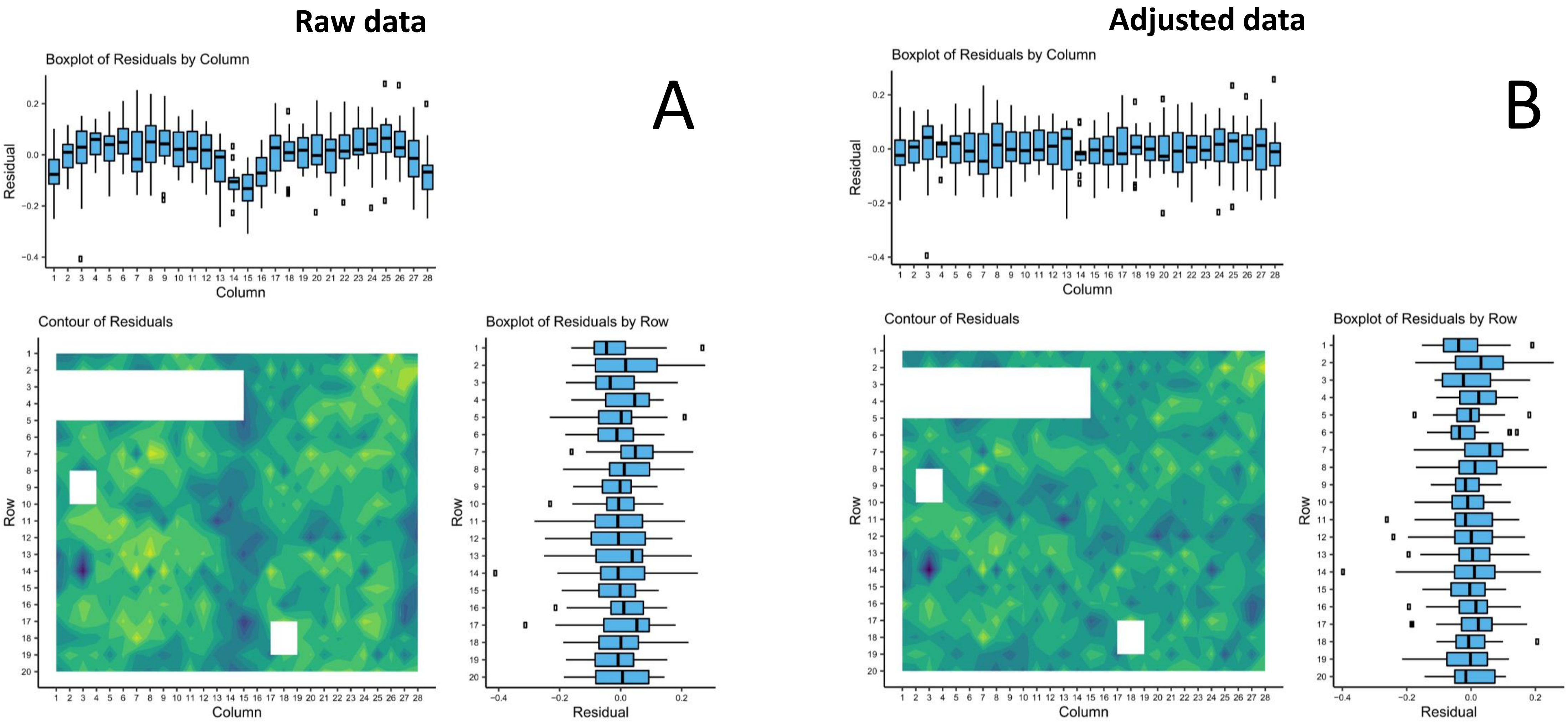

Additional File 3: Figure S13. A graphical representation of environmental variation within the controlled environment (CE) facility and the applied spatial adjustments. A: Raw data (prior to adjustments) which includes a heatmap for environmental variation across all positions within the CE facility as well as boxplots of the residuals to reflect the effect of environmental variations between rows and between columns for PH-W5. B: Adjusted data (after spatial adjustments) which includes a heatmap for environmental variation across all positions within the CE facility as well as boxplots of the residuals to reflect the effect of environmental variations between rows and between columns for PH-W5. Blank areas in the heatmaps represent missing data.

Plant Height- Week 6 (PH-W6)

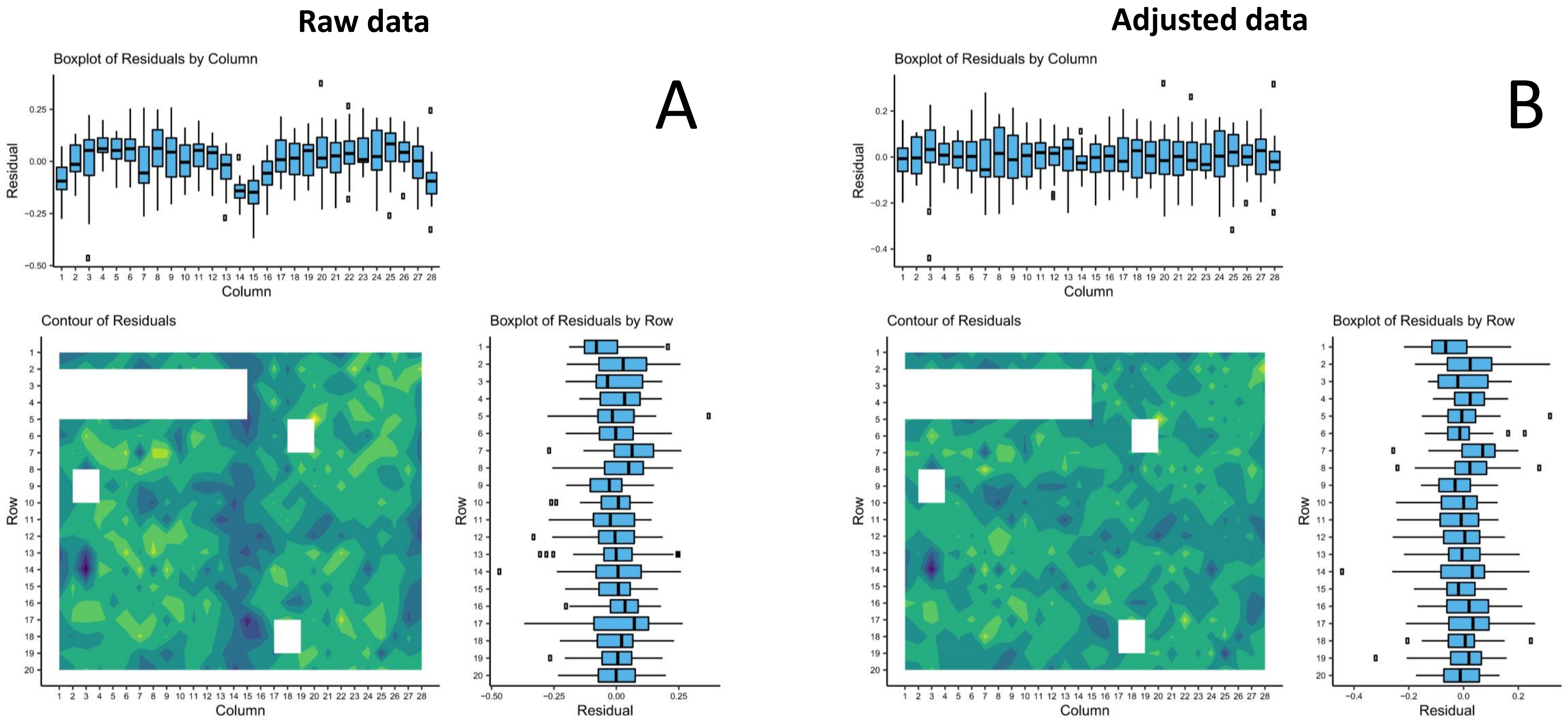

Additional File 3: Figure S14. A graphical representation of environmental variation within the controlled environment (CE) facility and the applied spatial adjustments. A: Raw data (prior to adjustments) which includes a heatmap for environmental variation across all positions within the CE facility as well as boxplots of the residuals to reflect the effect of environmental variations between rows and between columns for PH-W6. B: Adjusted data (after spatial adjustments) which includes a heatmap for environmental variation across all positions within the CE facility as well as boxplots of the residuals to reflect the effect of environmental variations between rows and between columns for PH-W6. Blank areas in the heatmaps represent missing data.

Plant Height- Week 7 (PH-W7)

Raw data **Adjusted data**

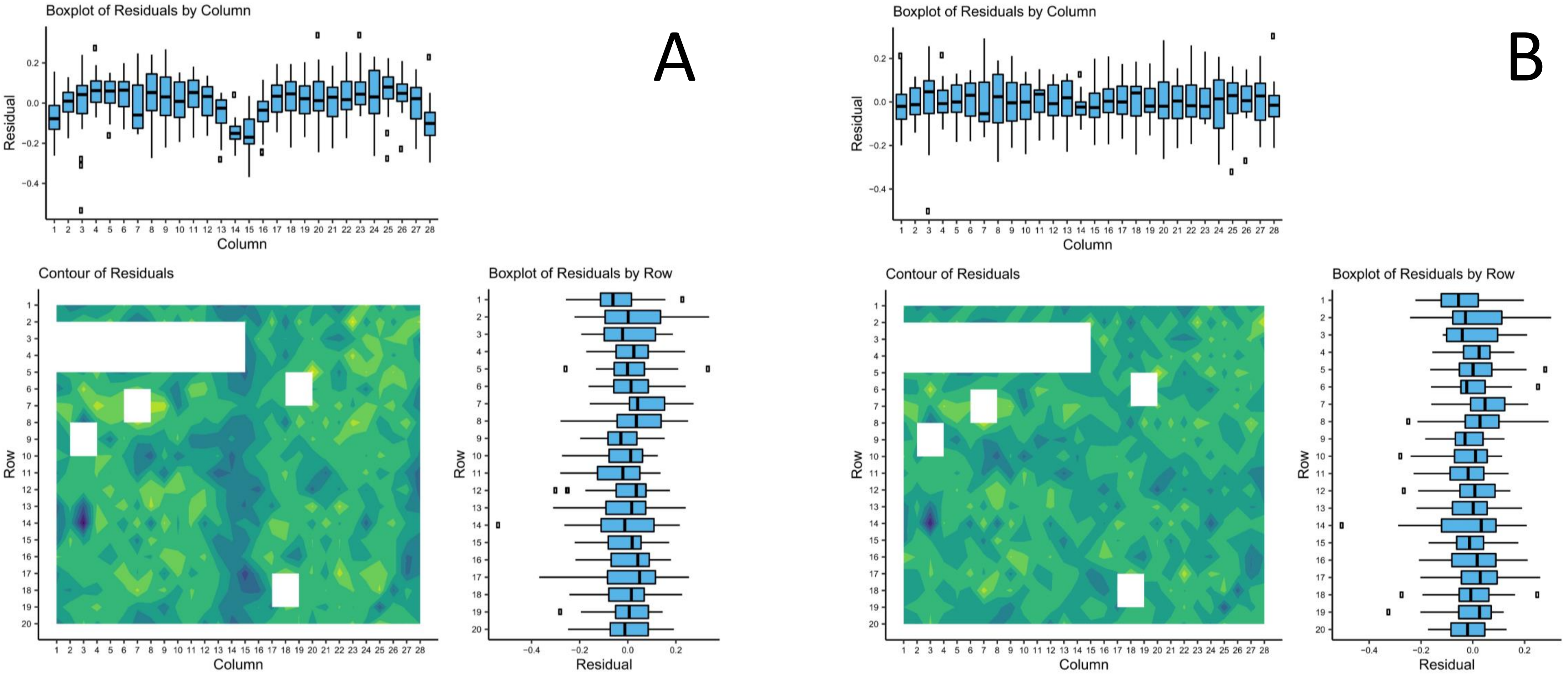

Additional File 3: Figure S15. A graphical representation of environmental variation within the controlled environment (CE) facility and the applied spatial adjustments. A: Raw data (prior to adjustments) which includes a heatmap for environmental variation across all positions within the CE facility as well as boxplots of the residuals to reflect the effect of environmental variations between rows and between columns for PH-W7. B: Adjusted data (after spatial adjustments) which includes a heatmap for environmental variation across all positions within the CE facility as well as boxplots of the residuals to reflect the effect of environmental variations between rows and between columns for PH-W7. Blank areas in the heatmaps represent missing data.

Plant Height- Week 8 (PH-W8)

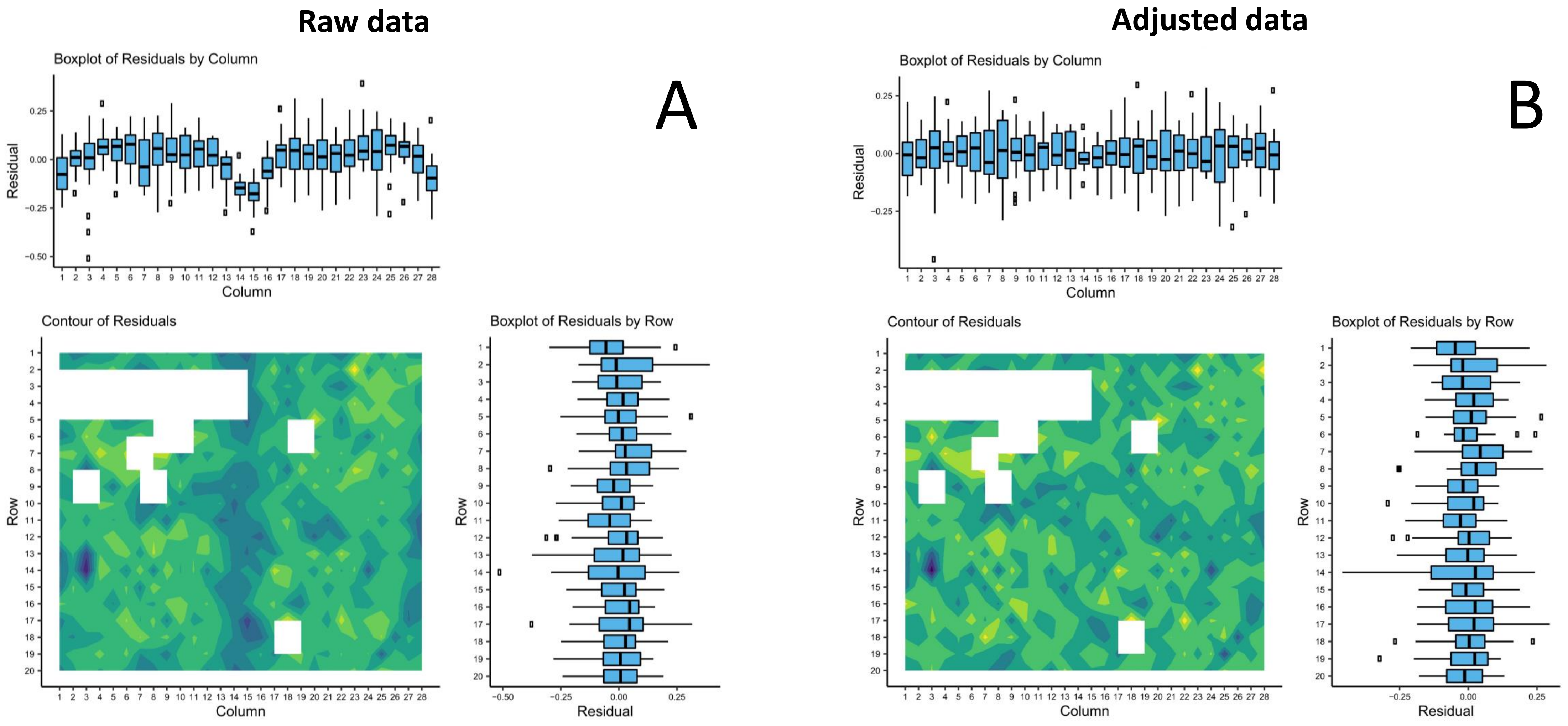

Additional File 3: Figure S16. A graphical representation of environmental variation within the controlled environment (CE) facility and the applied spatial adjustments. A: Raw data (prior to adjustments) which includes a heatmap for environmental variation across all positions within the CE facility as well as boxplots of the residuals to reflect the effect of environmental variations between rows and between columns for PH-W8. B: Adjusted data (after spatial adjustments) which includes a heatmap for environmental variation across all positions within the CE facility as well as boxplots of the residuals to reflect the effect of environmental variations between rows and between columns for PH-W8. Blank areas in the heatmaps represent missing data.

Plant Height- Week 9 (PH-W9)

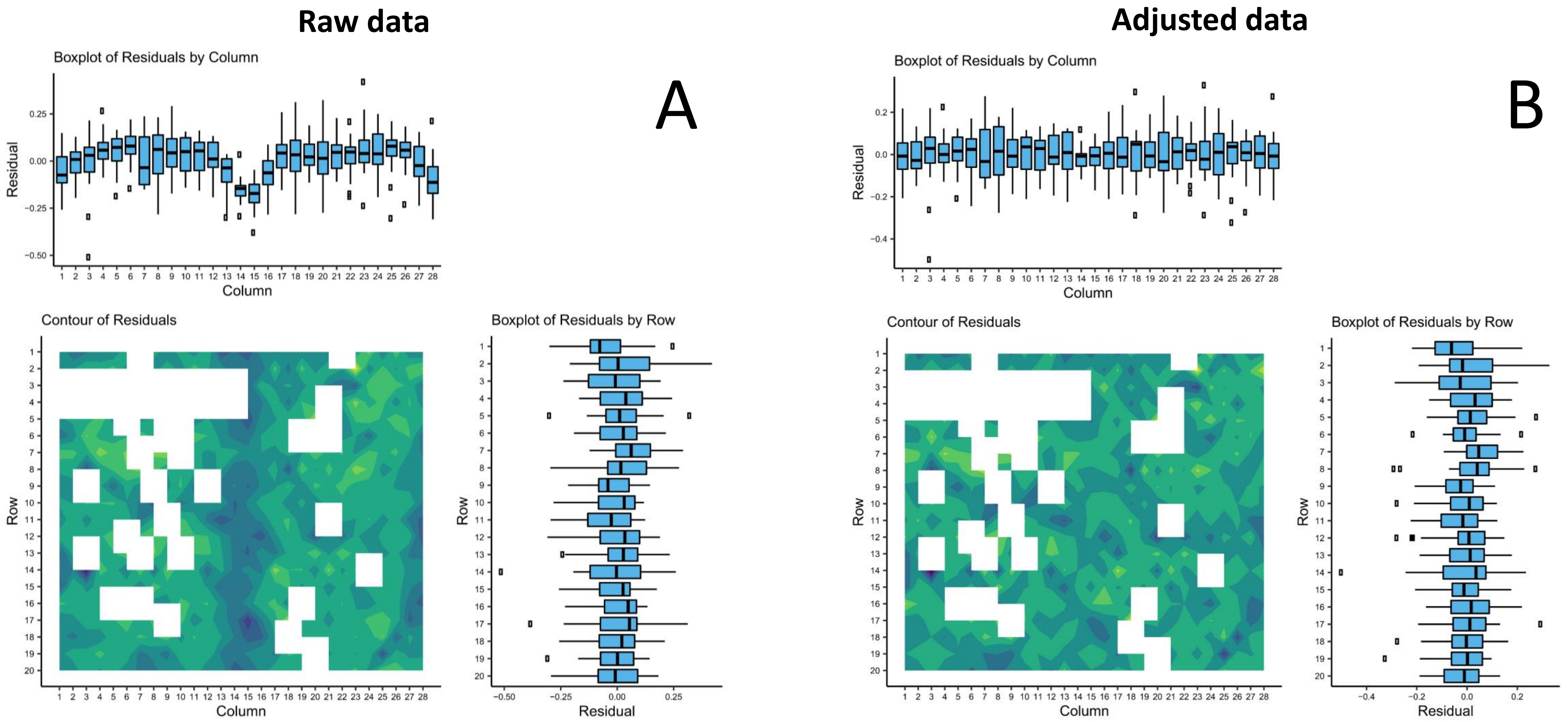

Additional File 3: Figure S17. A graphical representation of environmental variation within the controlled environment (CE) facility and the applied spatial adjustments. A: Raw data (prior to adjustments) which includes a heatmap for environmental variation across all positions within the CE facility as well as boxplots of the residuals to reflect the effect of environmental variations between rows and between columns for PH-W9. B: Adjusted data (after spatial adjustments) which includes a heatmap for environmental variation across all positions within the CE facility as well as boxplots of the residuals to reflect the effect of environmental variations between rows and between columns for PH-W9. Blank areas in the heatmaps represent missing data.

Stem Diameter- Week 2 (SD-W2)

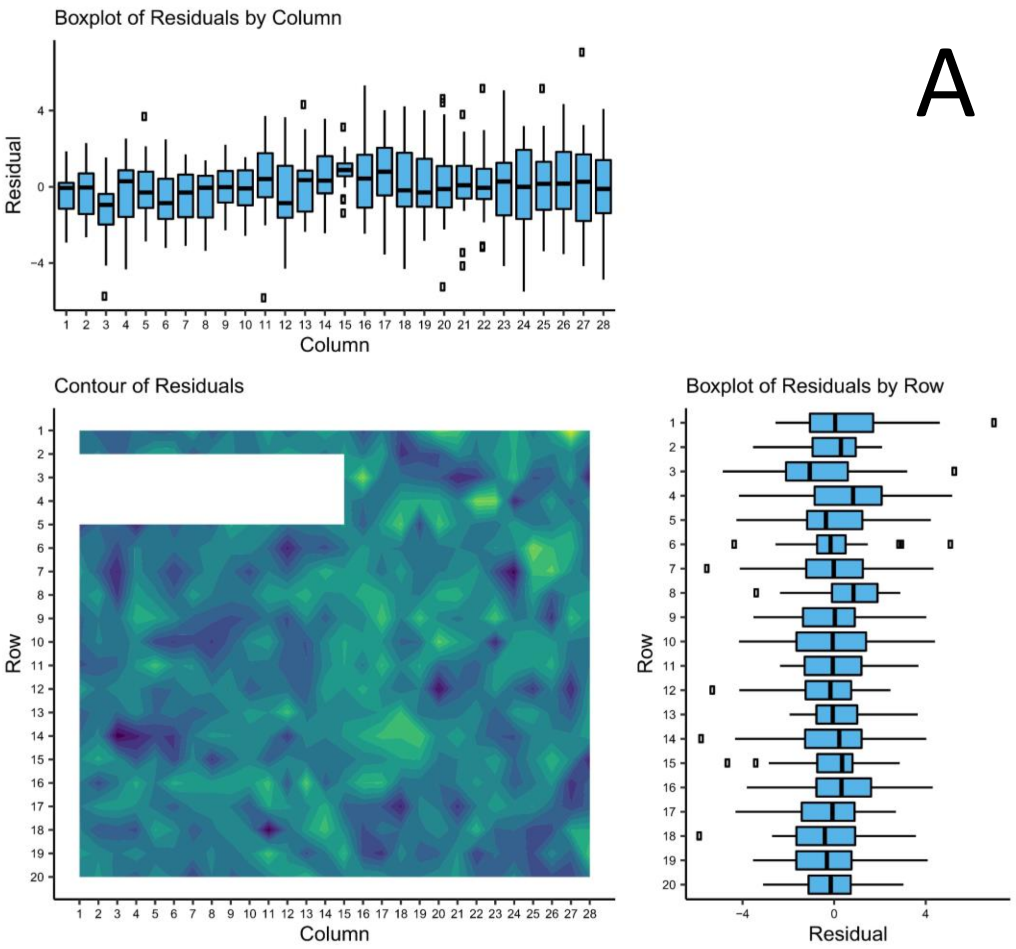

Additional File 3: Figure S18. A graphical representation of environmental variation within the controlled environment (CE) facility. Raw data is depicted via a heatmap for environmental variation across all positions within the CE facility as well as boxplots of the residuals to reflect the row and column position effect for SD-W2. Blank areas in the heatmaps represent missing data. Spatial adjustments were not required and therefore were not conducted.

Stem Diameter- Week 3 (SD-W3)

A

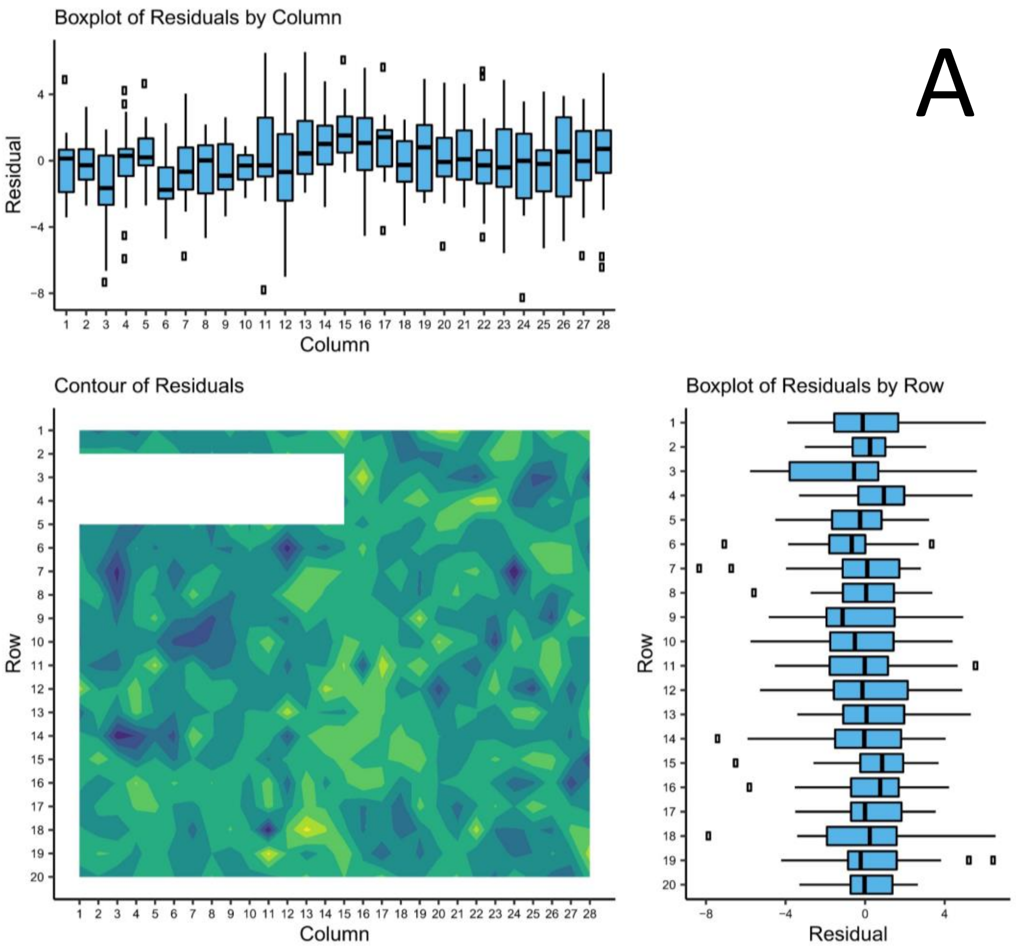

Additional File 3: Figure S19. A graphical representation of environmental variation within the controlled environment (CE) facility. Raw data is depicted via a heatmap for environmental variation across all positions within the CE facility as well as boxplots of the residuals to reflect the row and column position effect for SD-W3. Blank areas in the heatmaps represent missing data. Spatial adjustments were not required and therefore were not conducted.

Stem Diameter- Week 4 (SD-W4)

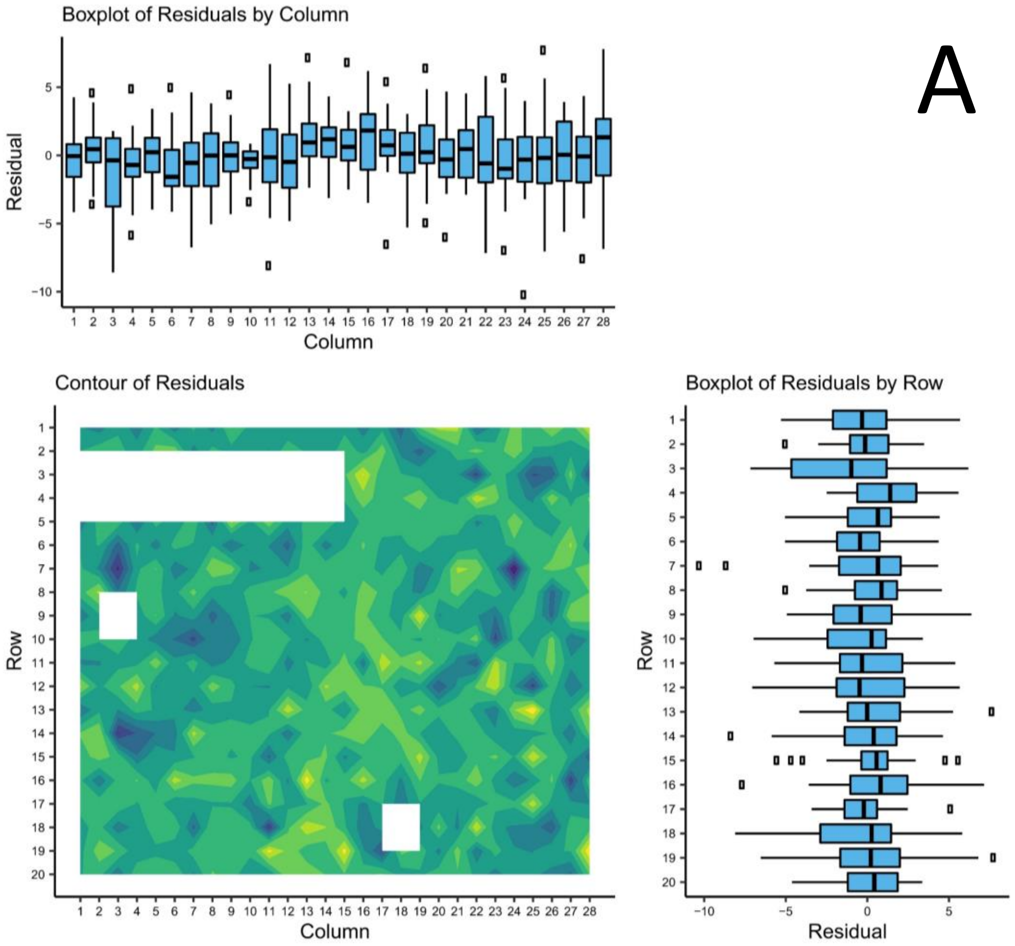

A

Additional File 3: Figure S20. A graphical representation of environmental variation within the controlled environment (CE) facility. Raw data is depicted via a heatmap for environmental variation across all positions within the CE facility as well as boxplots of the residuals to reflect the row and column position effect for SD-W4. Blank areas in the heatmaps represent missing data. Spatial adjustments were not required and therefore were not conducted.

Stem Diameter- Week 5 (SD-W5)

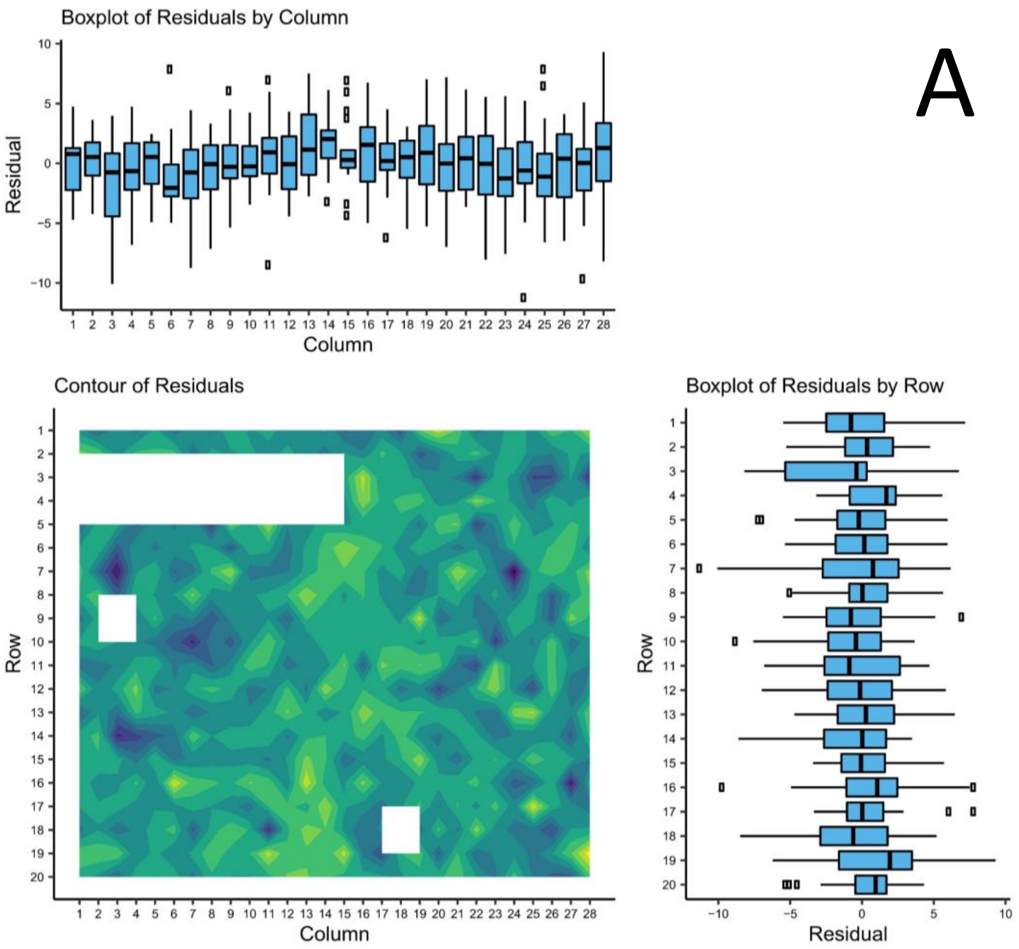

Additional File 3: Figure S21. A graphical representation of environmental variation within the controlled environment (CE) facility. Raw data is depicted via a heatmap for environmental variation across all positions within the CE facility as well as boxplots of the residuals to reflect the row and column position effect for SD-W5. Blank areas in the heatmaps represent missing data. Spatial adjustments were not required and therefore were not conducted.

Stem Diameter- Week 6 (SD-W6)

Raw data

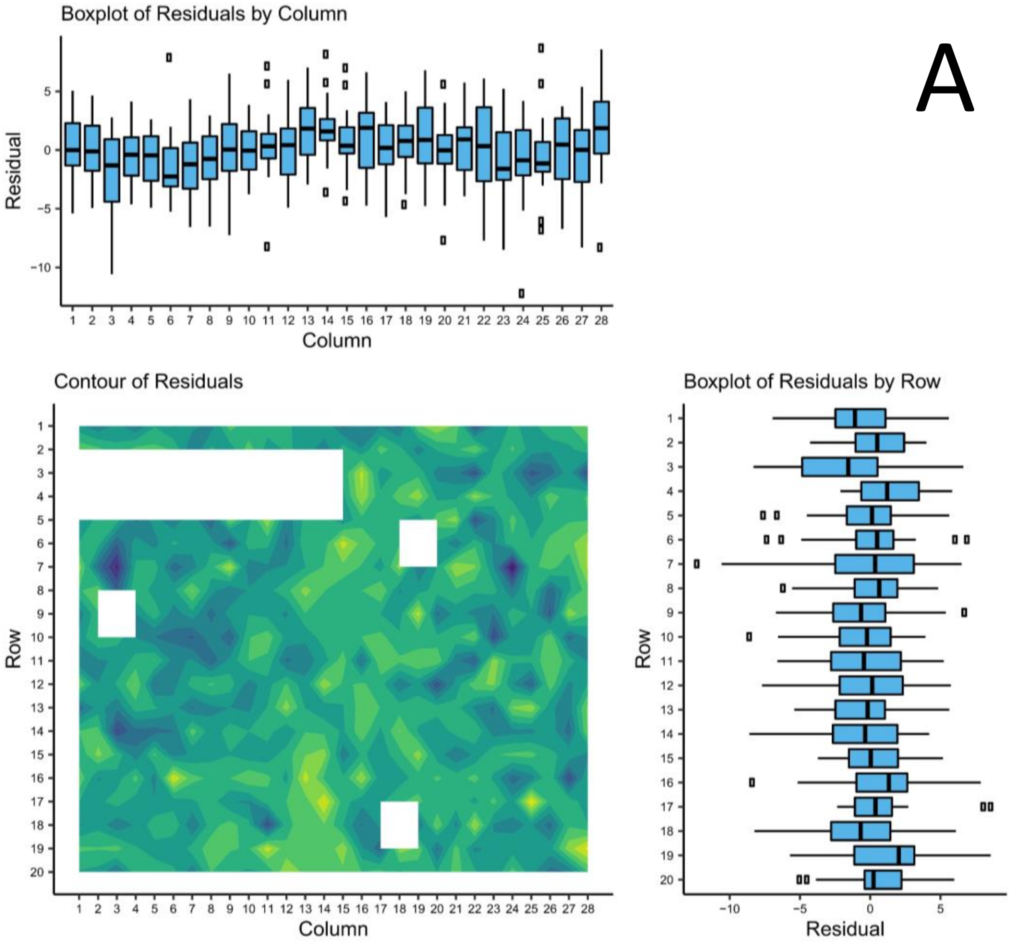

Adjusted data

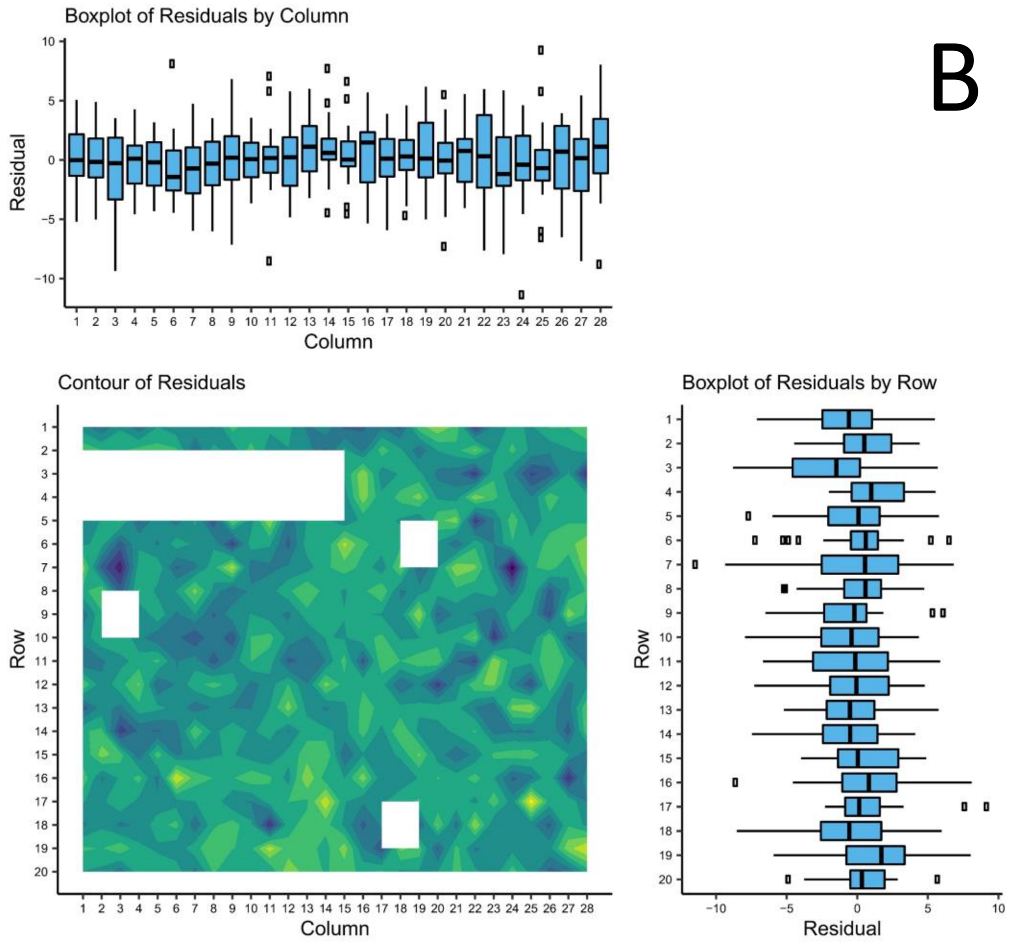

Additional File 3: Figure S22. A graphical representation of environmental variation within the controlled environment (CE) facility and the applied spatial adjustments. A: Raw data (prior to adjustments) which includes a heatmap for environmental variation across all positions within the CE facility as well as boxplots of the residuals to reflect the effect of environmental variations between rows and between columns for SD-W6. B: Adjusted data (after spatial adjustments) which includes a heatmap for environmental variation across all positions within the CE facility as well as boxplots of the residuals to reflect the effect of environmental variations between rows and between columns for SD-W6. Blank areas in the heatmaps represent missing data.

Stem Diameter- Week 7 (SD-W7)

Raw data | **Adjusted data**

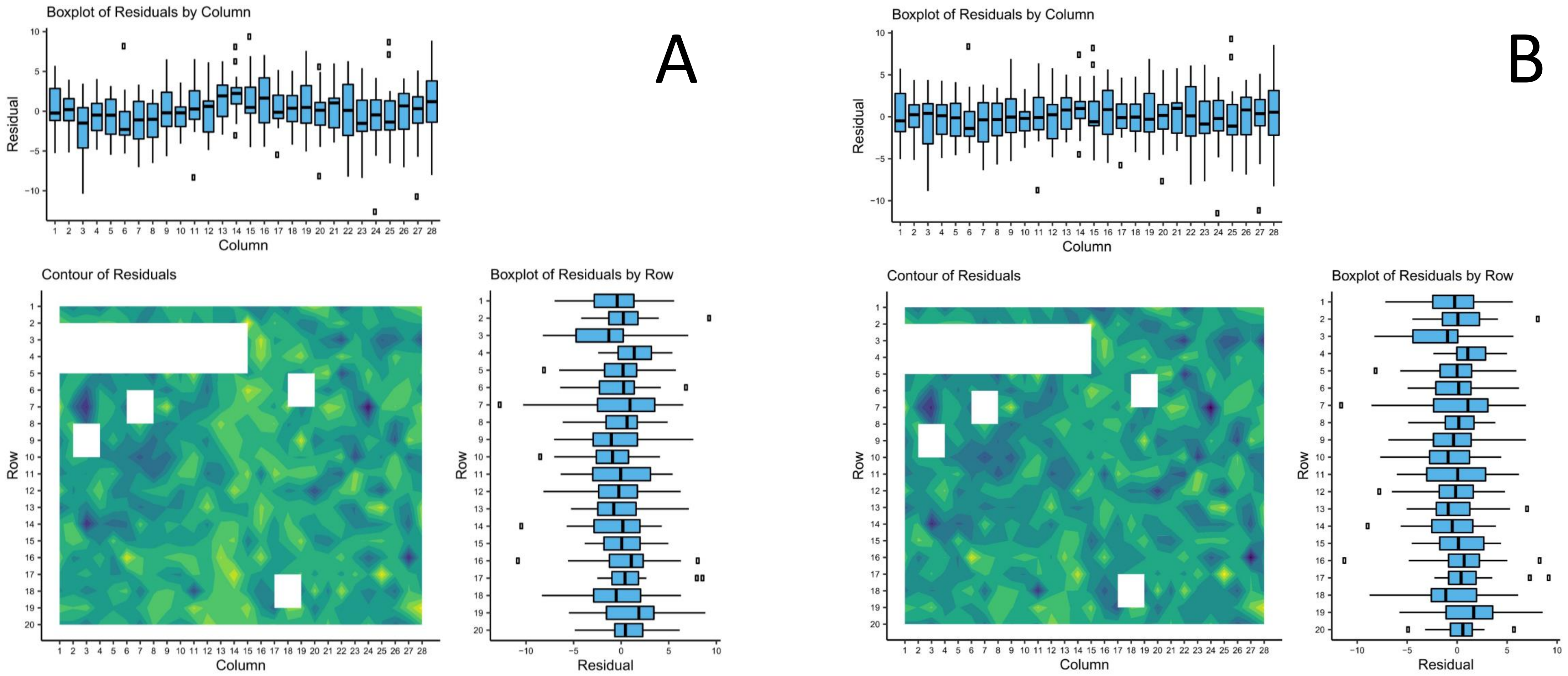

Additional File 3: Figure S23. A graphical representation of environmental variation within the controlled environment (CE) facility and the applied spatial adjustments. A: Raw data (prior to adjustments) which includes a heatmap for environmental variation across all positions within the CE facility as well as boxplots of the residuals to reflect the effect of environmental variations between rows and between columns for SD-W7. B: Adjusted data (after spatial adjustments) which includes a heatmap for environmental variation across all positions within the CE facility as well as boxplots of the residuals to reflect the effect of environmental variations between rows and between columns for SD-W7. Blank areas in the heatmaps represent missing data.

Stem Diameter- Week 8 (SD-W8)

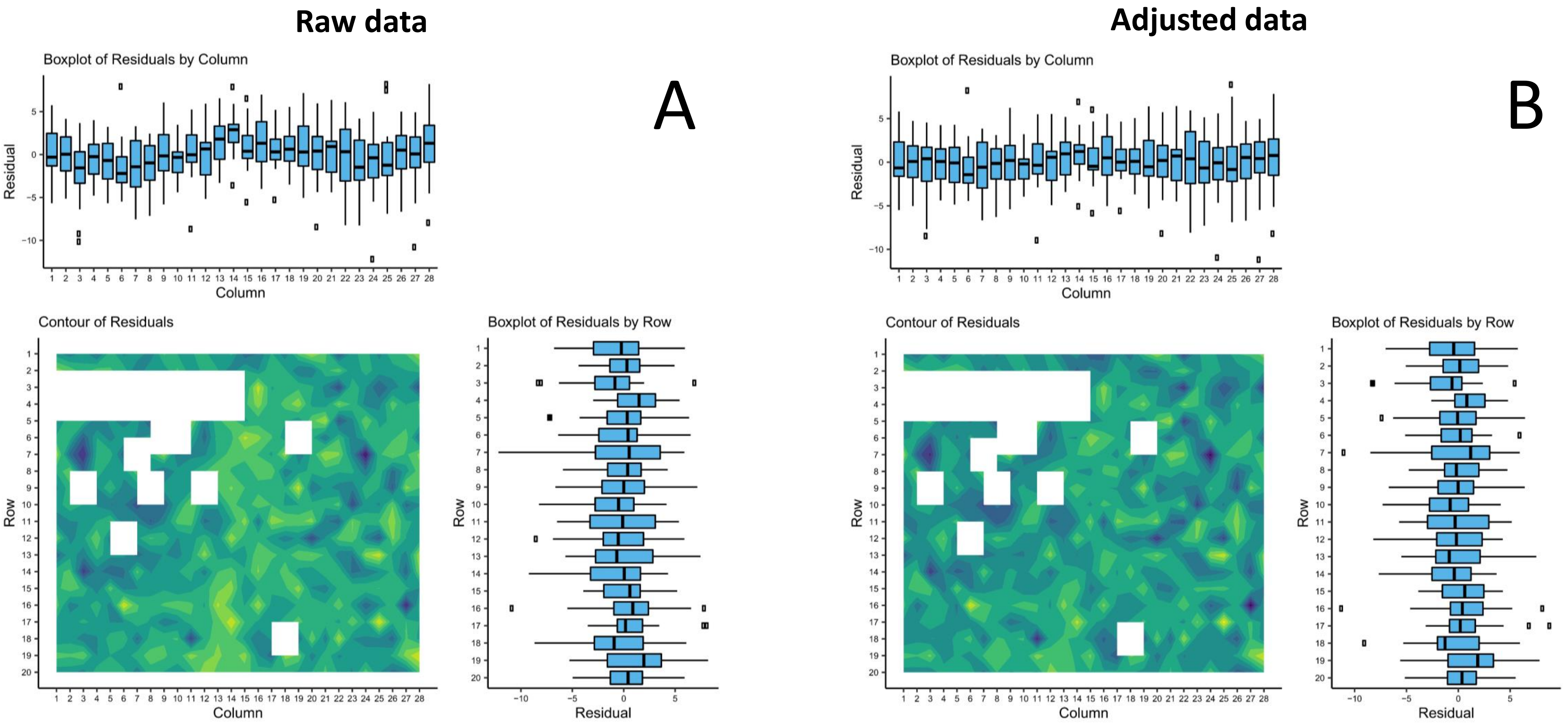

Additional File 3: Figure S24. A graphical representation of environmental variation within the controlled environment (CE) facility and the applied spatial adjustments. A: Raw data (prior to adjustments) which includes a heatmap for environmental variation across all positions within the CE facility as well as boxplots of the residuals to reflect the effect of environmental variations between rows and between columns for SD-W8. B: Adjusted data (after spatial adjustments) which includes a heatmap for environmental variation across all positions within the CE facility as well as boxplots of the residuals to reflect the effect of environmental variations between rows and between columns for SD-W8. Blank areas in the heatmaps represent missing data.
