# Supplementary material for: The characterization of key physiological traits of medicinal cannabis (Cannabis sativa L.) as a tool for precision breeding
Source: BMC Plant Biol. 2021 Jun 26;21:294. doi: 10.1186/s12870-021-03079-2 (PMC8235858; doi:10.1186/s12870-021-03079-2)
Supplement: Supplementary file 4 — Additional file 4: The determination of replicate quantities. [file 12870_2021_3079_MOESM4_ESM.docx]

Additional File 4: The determination of replicate quantities

In a preliminary study, 20 clonal replicates of 2 genotypes were cultivated and examined under CE facility (the same facility that was later used for the main trial). On harvest day, plants height was measured, and the mean and standard deviation of each genotype were calculated. for each genotype, a normal distribution was constructed in order to assess the minimum number of experimental replicates that will reduce the probability of sample error and accurately reflect plant height population mean.

According to the genotypic normal distribution and in accordance with probability considerations, random entries were cyclically sampled. The number of data points sampled in each cycle ranged from 1 to 20 in order to simulate possible numbers of plant repetition. Sampling cycles repeated 1000 times and the mean of the entries drawn within each cycle was calculated (black bubbles on Figures S1 and S2). Sampling cycles mean (blue line on Figures S1 and S2) and 2 levels of standard deviation (Figures S1 and S2, purple ribbon indicates 1 standard deviation and grey ribbon indicates 2 standard deviations) were calculated for each replicates number. Accordingly, the examination of each replicate entry identified 4 to 5 clonal replicates to be the minimum number of repetitions that can significantly reduce the probability of measurement errors and accurately reflect the genotypic mean.


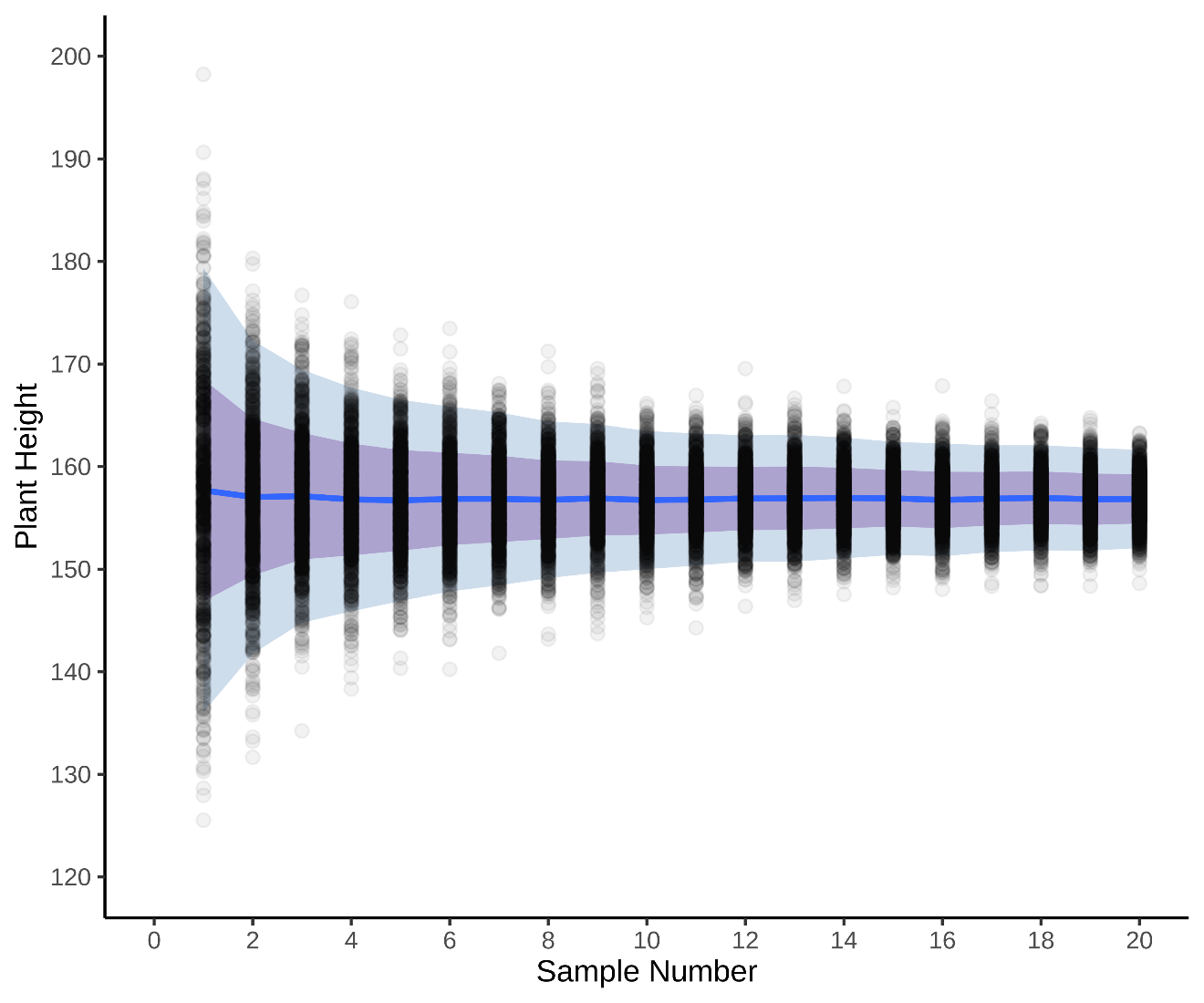


Additional File 4: Figure S1. The distribution of plant height means and standard deviation under different replicate quantities for genotype 1 (genotype ID: 445146).


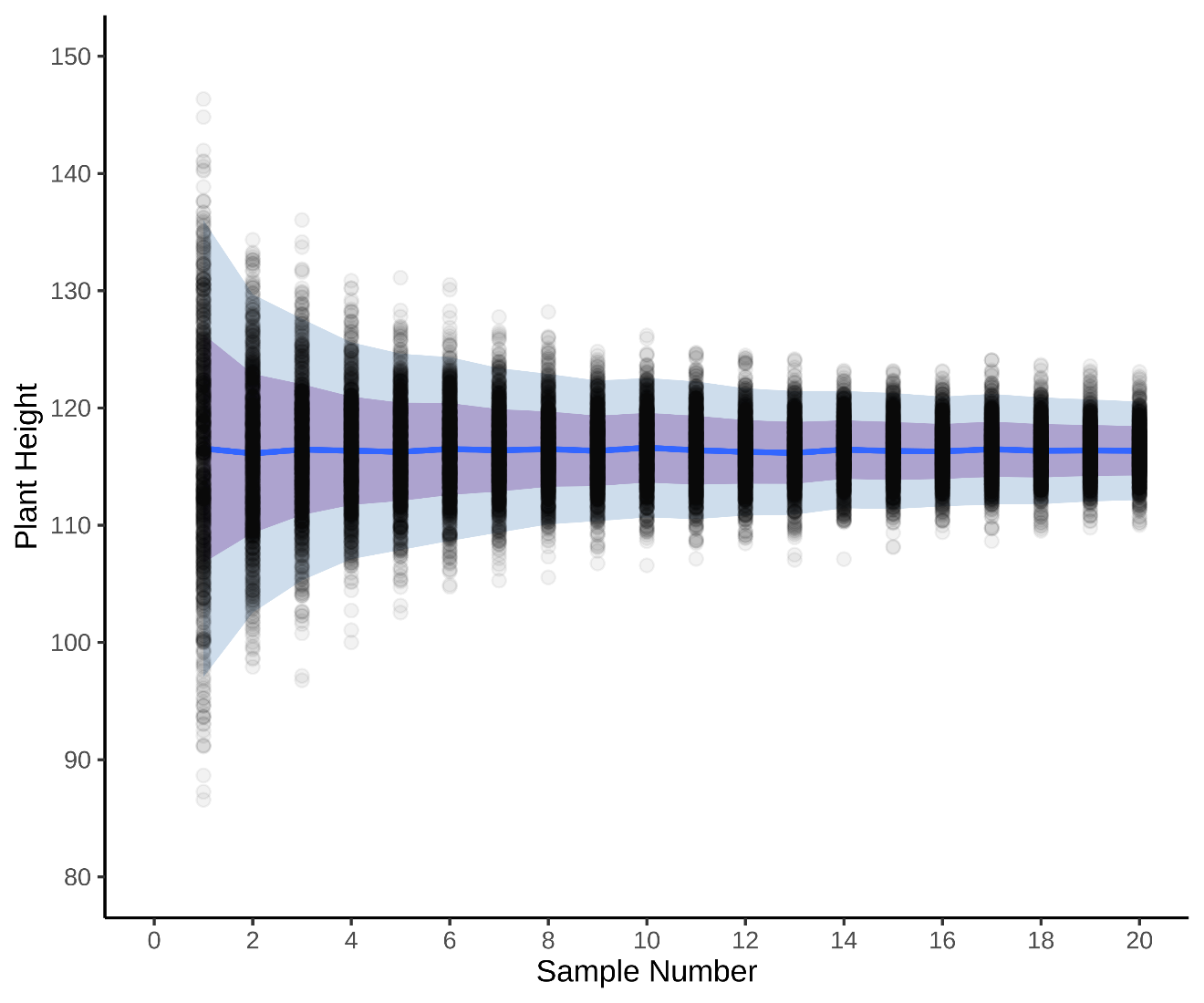


Additional File 4: Figure S2. The distribution of plant height means and standard deviation under different replicate quantities for genotype 2 (genotype ID: 446020).
