# Supplementary material for: The characterization of key physiological traits of medicinal cannabis (Cannabis sativa L.) as a tool for precision breeding
Source: BMC Plant Biol. 2021 Jun 26;21:294. doi: 10.1186/s12870-021-03079-2 (PMC8235858; doi:10.1186/s12870-021-03079-2)
Supplement: Supplementary file 5 — Additional file 5: Figure S1 Trial design and spatial arrangement. Black boxes represent rolling benches, red and blue lines represent room rows and room columns, respectively and green numbers indicate the first and last plot on each bench. [file 12870_2021_3079_MOESM5_ESM.pptx]

## Slide 1
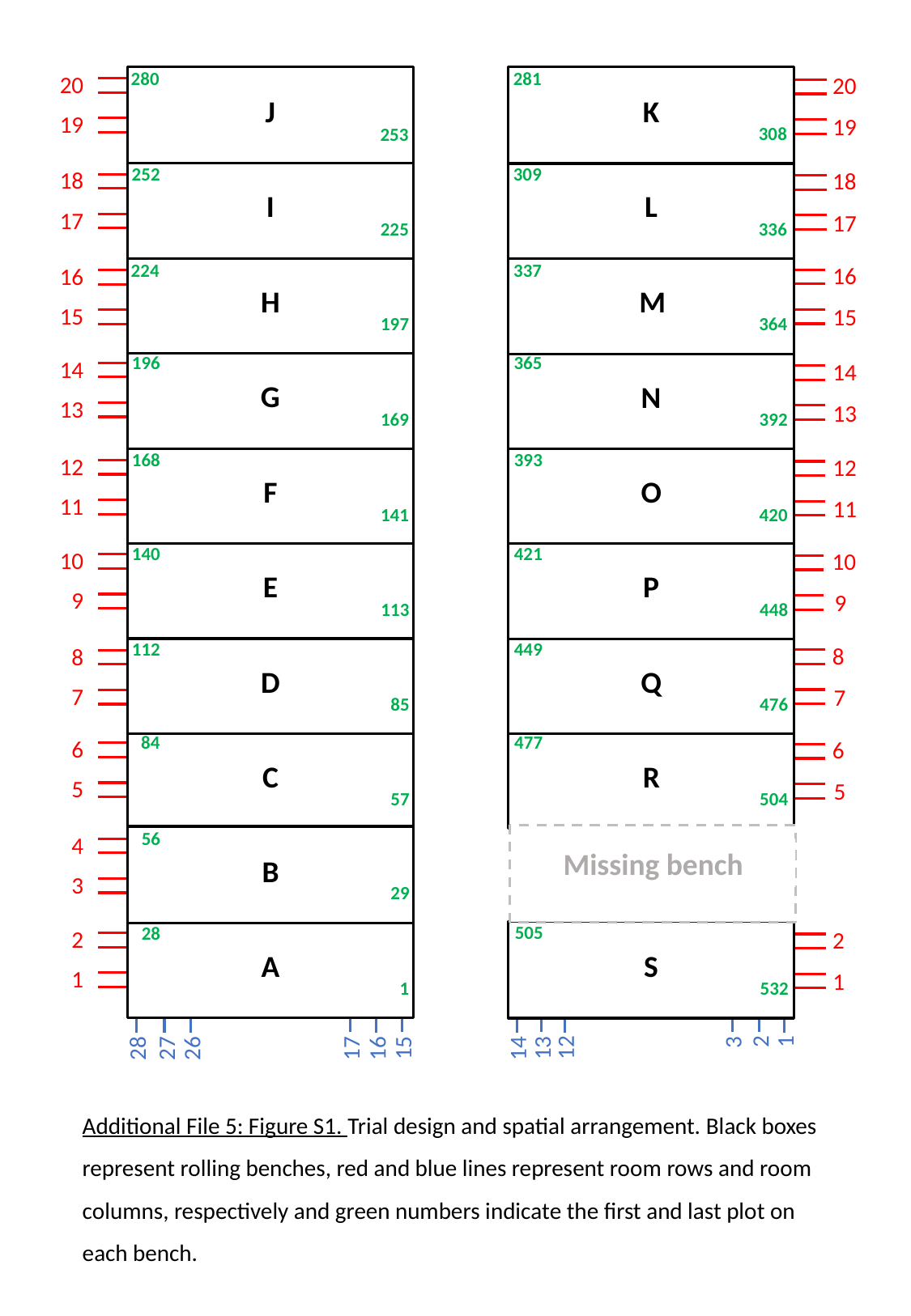

281
309
337
365
393
421
449
477
505
280
252
224
196
168
140
112
84
56
28
20
19
18
17
16
15
14
13
12
11
10
9
8
7
6
5
4
3
2
1
20
19
18
17
16
15
14
13
12
11
10
9
8
7
6
5
2
1
J
K
308
336
364
392
420
448
476
504
532
253
225
197
169
141
113
85
57
29
1
I
L
H
M
G
N
F
O
E
P
D
Q
C
R
Missing bench
B
A
S
1
2
3
12
13
15
26
28
27
14
17
16
Additional File 5: Figure S1. Trial design and spatial arrangement. Black boxes represent rolling benches, red and blue lines represent room rows and room columns, respectively and green numbers indicate the first and last plot on each bench.
